# A new insect trackway from the Upper Jurassic—Lower Cretaceous eolian sandstones of São Paulo State, Brazil: implications for reconstructing desert paleoecology

**DOI:** 10.7717/peerj.8880

**Published:** 2020-05-22

**Authors:** Bernardo de C.P. e M. Peixoto, M. Gabriela Mángano, Nicholas J. Minter, Luciana Bueno dos Reis Fernandes, Marcelo Adorna Fernandes

**Affiliations:** 1 Laboratório de Paleoicnologia e Paleoecologia, Departamento de Ecologia e Biologia Evolutiva, Universidade Federal de São Carlos (UFSCar), São Carlos, São Paulo, Brazil; 2 Programa de Pós Graduação em Ecologia e Recursos Naturais, Centro de Ciências Biológicas e da Saúde, Universidade Federal de São Carlos (UFSCar), São Carlos, São Paulo, Brazil; 3 Department of Geological Sciences, University of Saskatchewan, Saskatoon, Saskatchewan, Canada; 4 School of the Environment, Geography, and Geosciences, University of Portsmouth, Portsmouth, Hampshire, United Kingdom

**Keywords:** Deserts, *Erg*, Palaeoecology, Trophic web, Gondwana, Ichnofacies, Botucatu Formation, Paraná Basin

## Abstract

The new ichnospecies *Paleohelcura araraquarensis* isp. nov. is described from the Upper Jurassic-Lower Cretaceous Botucatu Formation of Brazil. This formation records a gigantic eolian sand sea (*erg*), formed under an arid climate in the south-central part of Gondwana. This trackway is composed of two track rows, whose internal width is less than one-quarter of the external width, with alternating to staggered series, consisting of three elliptical tracks that can vary from slightly elongated to tapered or circular. The trackways were found in yellowish/reddish sandstone in a quarry in the Araraquara municipality, São Paulo State. Comparisons with neoichnological studies and morphological inferences indicate that the producer of *Paleohelcura araraquarensis* isp. nov. was most likely a pterygote insect, and so could have fulfilled one of the ecological roles that different species of this group are capable of performing in dune deserts. The producer could have had a herbivorous or carnivorous diet or been part of the fauna of omnivores, being able to adopt herbivorous, carnivorous, and saprophagous diets when necessary. In modern dune deserts, some species of pterygote insects are detritivores (like Tenebrionidae), relying on organic matter that accumulated among the sand grains of the dunes during dry periods with no plant growth. The presence of additional burrows suggests that the Botucatu paleodesert would have had a detritivorous fauna like this. Based on the interpretation of the ichnofossil producers, it was possible to reconstruct the food web of this paleodesert. All the omnivorous and herbivorous invertebrates and the herbivorous ornithopod dinosaurs made up the primary consumers. These animals were, in turn, the food source for bigger carnivorous or omnivorous animals unable to feed on detritus, like arachnids, possible predatory insects, mammaliaforms, and theropod dinosaurs. The highest trophic level was occupied by larger theropod dinosaurs and mammaliaforms, which, because of their size, could prey upon a wide range of animals. The producer of *Paleohelcura araraquarensis* isp. nov. could have been a primary consumer if it were an omnivorous detritivore or a herbivore, or a secondary consumer if it were produced by a predatory insect or an omnivore relying on animal biomass. The description of this new trackway expands the knowledge on the faunal composition of the Botucatu paleodesert and provides insights into the ecological relationships in ancient deserts. The presence of these arthropod trackways in Mesozoic eolian deposits helps to trace a continuity between Paleozoic and post-Paleozoic desert ichnofaunas, further reinforcing a single *Octopodichnus—Entradichnus* Ichnofacies for eolian deposits.

## Introduction

The Botucatu Formation, a stratigraphic unit of the Paraná Basin, is the testament of a gigantic sand desert (*erg*) that existed from the Late Jurassic to the Early Cretaceous in the south-central part of the supercontinent Gondwana, totaling an area of 1.5 × 10^6^ km^2^, encompassing parts of Brazil, Argentina, Uruguay, Paraguay, Namibia and South Africa (Scherer & Goldberg, 2007). Ichnofossils are the only evidence of animal life in this ancient desert because no animal body fossils have been found. Therefore, trace fossils play a central role in understanding animal diversity and ecological relationships in this ancient erg.

Eolian deposits have been traditionally considered of minor interest from an ichnologic perspective. However, this situation has changed at an accelerated rate in recent years with the publication of several papers on the topic (e.g.,  Ekdale, Bromley & Loope, 2007; Ekdale & Bromley, 2012; Good & Ekdale, 2014; Krapovickas et al., 2016; Carmona, Ponce & Wetzel, 2018; Xing et al., 2018; Buatois & Echevarría, 2019; Marchetti et al., 2019a; Marchetti et al., 2019b). A recent review emphasized the complex pattern of trace-fossil distribution in eolian and related facies, the debate surrounding definition of an archetypal ichnofacies, and delineation of macroevolutionary trends in desert environments (Krapovickas et al., 2016). Documentation of trace fossils in desert successions is of paramount importance to provide support to these models, and to help clarify the diagnostic characteristics of the so-called *Octopodichnus-Entradichnus* Ichnofacies.

Several trackways of tetrapods and arthropods have been recovered from eolian dune deposits of different ages (McKeever, 1991, table 1). There has been contention over the preservation potential of such trackways in ‘dry’ dune deposits and whether they had to have been produced subaqueously (Brand, 1979; Brand & Tang, 1991; McKeever, 1991; Brand, 1992; Lockley, 1992; Loope, 1992). Moisture (Mckee, 1947; Sadler, 1993) and the presence of clay minerals (Loope, 1986; McKeever, 1991) between sand grains have both been proposed to play a role in trackway stabilization and preservation potential. Experiments have demonstrated that the combination of the two can lead to enhanced survivorship of arthropod trackways over those made in dry sand or sand with just surface moisture or the presence of clay minerals alone (Davis, Minter & Braddy, 2007). Nevertheless, it has been recently argued that special conditions are not necessarily needed to preserve such trace fossils (see Davies & Shillito, 2018).

**Table 1:** Occurrence of invertebrate ichnofossils from the Botucatu Formation and the first references describing them.

Locality	Ichnofossil Description	Reference
Quarry 3–4 km from São Carlos (SP) (probably Migliato or Araújo quarry)	“Worm Tunnels”	Pacheco & De Amaral (1913)
Sierra of Botucatu (SP)	“Worm tubes.”	Almeida (1954)
Pacaembú neighborhood, São Carlos (SP)	“Worm trails.”	Bjornberg & Tolentino (1959)
São Tomás Ranch quarry, Ibaté Municipality (SP)	“Fossil tracks of conchostracans(?)”	Paraguassu (1970)
Quarry near Araraquara (SP)	“Vermiform trails and tracks of arthropods.”	Leonardi (1980)
São Bento Quarry, Araraquara (SP)	“Arthropods trackways”	(Leonardi, 1984)
	“Arthropod trails”	(Leonardi & Sarjeant, 1986)
	“Invertebrate trackways and burrows”	(Leonardi & Godoy, 1980)
	(...)Ten rare forms of invertebrate trails, mainly attributable to arthropods (arachnids and insects, adults or larvae)(...)	(Leonardi, Carvalho de & Fernandes, 2007)
	Insects, scorpions and spiders	(Fernandes, 2005).
Itaguaçu Farm Quarry, São Carlos (SP)	“Trails of vermiform invertebrates.”	Leonardi & Godoy (1980)
Campo Minado Cave, Sierra of Itaqueri, Ipeúna (SP)	*Taenidium serpentinum* and *Skolithos linearis*	Peixoto et al. (2016)
Sobradinho Farm, Araguari (MG)	Burrows of xylophagous termites and Coleoptera insects in conifer wood.	Riff, Kloster & Riff (2017)

**Notes.**

SP São Paulo State (Brazil) MG Minas Gerais State (Brazil)

Ichnofossils descriptions were translated literally from the original sources. Modified from Leonardi, Carvalho de & Fernandes (2007).

The aims of this study are to: (i) describe the new ichnospecies, *Paleohelcura araraquarensis* isp. nov., which consists of trackways produced by pterygote insects walking on sand dunes; (ii) discuss the implications of this record with respect to ecological relationships within the Botucatu paleodesert; and (iii) assess its importance for our understanding of eolian dune ichnofacies.

### Previous work

Most of the previous studies in the Botucatu Formation focused on tetrapod trackways, with only two publications dealing in detail with invertebrate trace fossils (Fernandes, Netto & Carvalho de, 1988; Fernandes, Carvalho & Netto, 1990). Before these studies, invertebrate trackways were only mentioned within the context of vertebrate ichnofaunas as the source of food for the presumed mammaliaform producer of *Brasilichnium elusivum* (Leonardi, 1981, p. 803). Subsequently, Leonardi (1984, p. 54) illustrated invertebrate trackways identical to those documented in this study, but whose repository is unknown. Arthropod trackways from São Bento Quarry (Araraquara city—São Paulo State) were later illustrated as well by Leonardi & Sarjeant (1986, p. 83), but no further information regarding a repository was provided. Leonardi, Carvalho de & Fernandes (2007) reported trace fossils of insects and arachnids in Araraquara (São Bento Quarry), but no illustrations were provided. Fernandes (2005) identified arachnid tracks in slabs from São Bento Quarry, and interpreted them as made by scorpions and spiders. Peixoto et al. (2016) documented new findings of *Taenidium serpentinum* and *Skolithos linearis*, probably produced by insects. The occurrences of invertebrate ichnofossils are summarized in Table 1.

The only plant fossils from the Botucatu Formation are conifer trunks, found in the region of Araguari (Minas Gerais State), north of the Tringulo Mineiro, within the limits of the sandstone occurrence area of this unit (Pires et al., 2011; Malaquias, Riff & Riff, 2017). Those trunks exhibit xylophagous marks assigned to termites (Isoptera) and beetles (Coleoptera) (Riff, Kloster & Riff, 2017).

With respect to the vertebrate trace-fossil record of the Botucatu Formation, there are two ichnospecies of *Brasilichnium* produced by small mammaliaform organisms: one demonstrating cursorial locomotion described as *B. elusivum* (Leonardi, 1981; Fernandes & Carvalho, 2008), and the other one in hopping locomotion (D’Orazi Porchetti, Bertini & Langer, 2017a), described as *B. saltatorium* (Buck et al., 2017b). There is also a record of a burrow compatible with the *Brasilichnium elusivum* producer (Manes, Da Silva & Scheffler, 2017). There is some controversy in describing new ichnotaxa based on differences in locomotion patterns instead of objective morphological attributes of the footprints alone (Lockley, 2007; Minter, Braddy & Davis, 2007). Nevertheless, the presence of a hopping behavior (e.g., *B. saltatorium*) is useful in indicating a biomechanical capability that constrains eligible clades of possible producers, together with when this biomechanical capability appeared.

Trackways of mammaliaforms larger than the producer of *Brasilichnium elusivum* have been described independently as *Brasilichnium anaitti* (D’Orazi Porchetti, Bertini & Langer, 2017b) and as *Aracoaraichnium leonardii* (Buck et al., 2017a). These two ichnotaxa bear several morphological similarities and were described from slabs reposited in different scientific collections. In addition, theropod and ornithopod dinosaur trackways have been recorded (Leonardi, 1979; Leonardi, 1980; Leonardi & Godoy, 1980; Leonardi, 1981; Leonardi, 1984; Leonardi & Sarjeant, 1986; Leonardi, 1987; Leonardi et al., 2002; Fernandes, 2005; Leonardi, Carvalho de & Fernandes, 2007; Francischini et al., 2015). Also noteworthy is the rare occurrence of an urolite, a biogenic mark interpreted as the result of the liquid extrusion of urine from dinosaurs onto unconsolidated sediment (Fernandes, Fernandes & Souto, 2004).

### Geological setting

**Figure 1:**
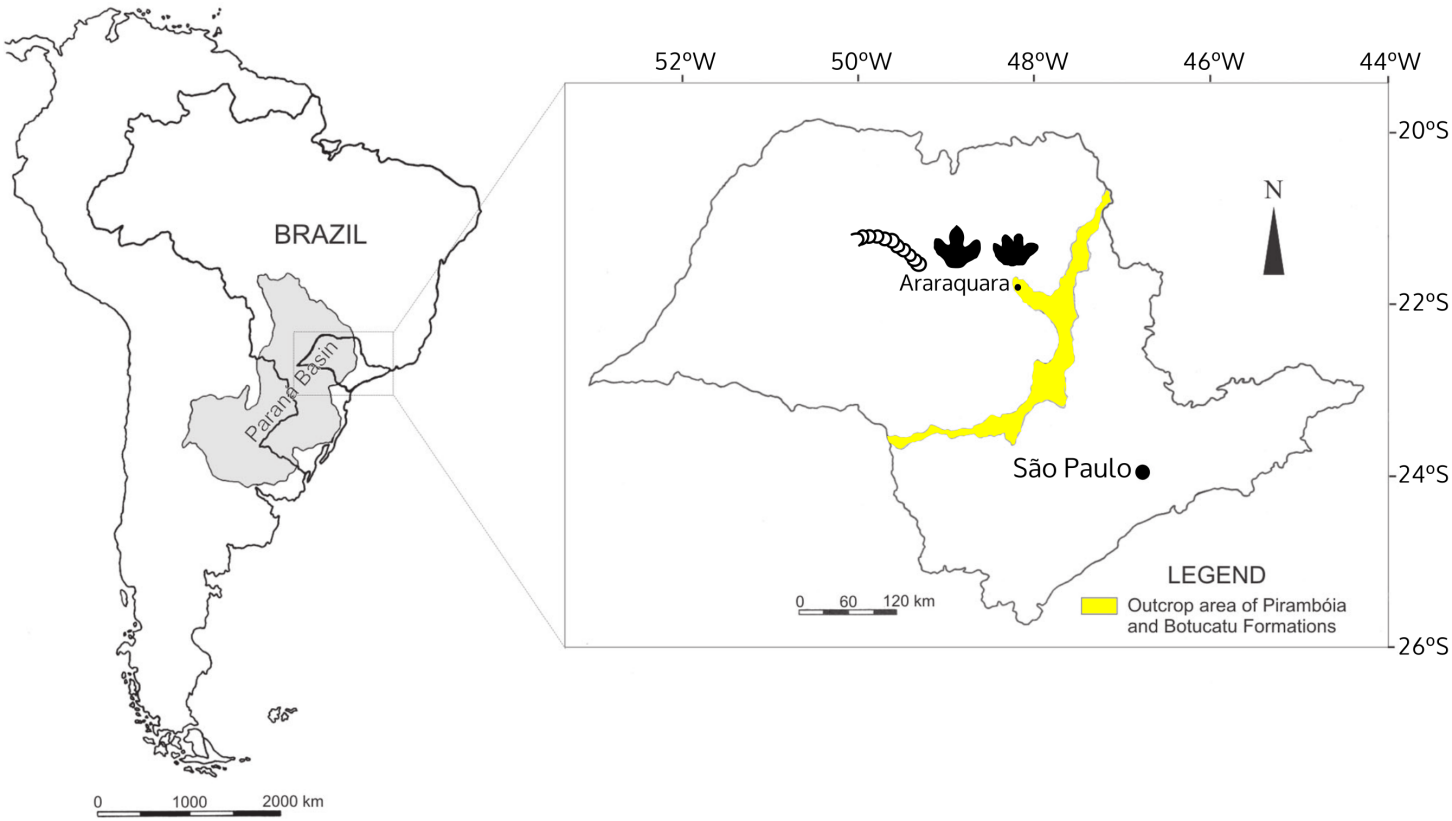
The localization of Araraquara City, where the fossils were collected, and outcrop area of the Botucatu and Pirambóia formations in the State of São Paulo, Brazil. Modified from Fernandes, Fernandes & Souto (2004).

**Figure 2:**
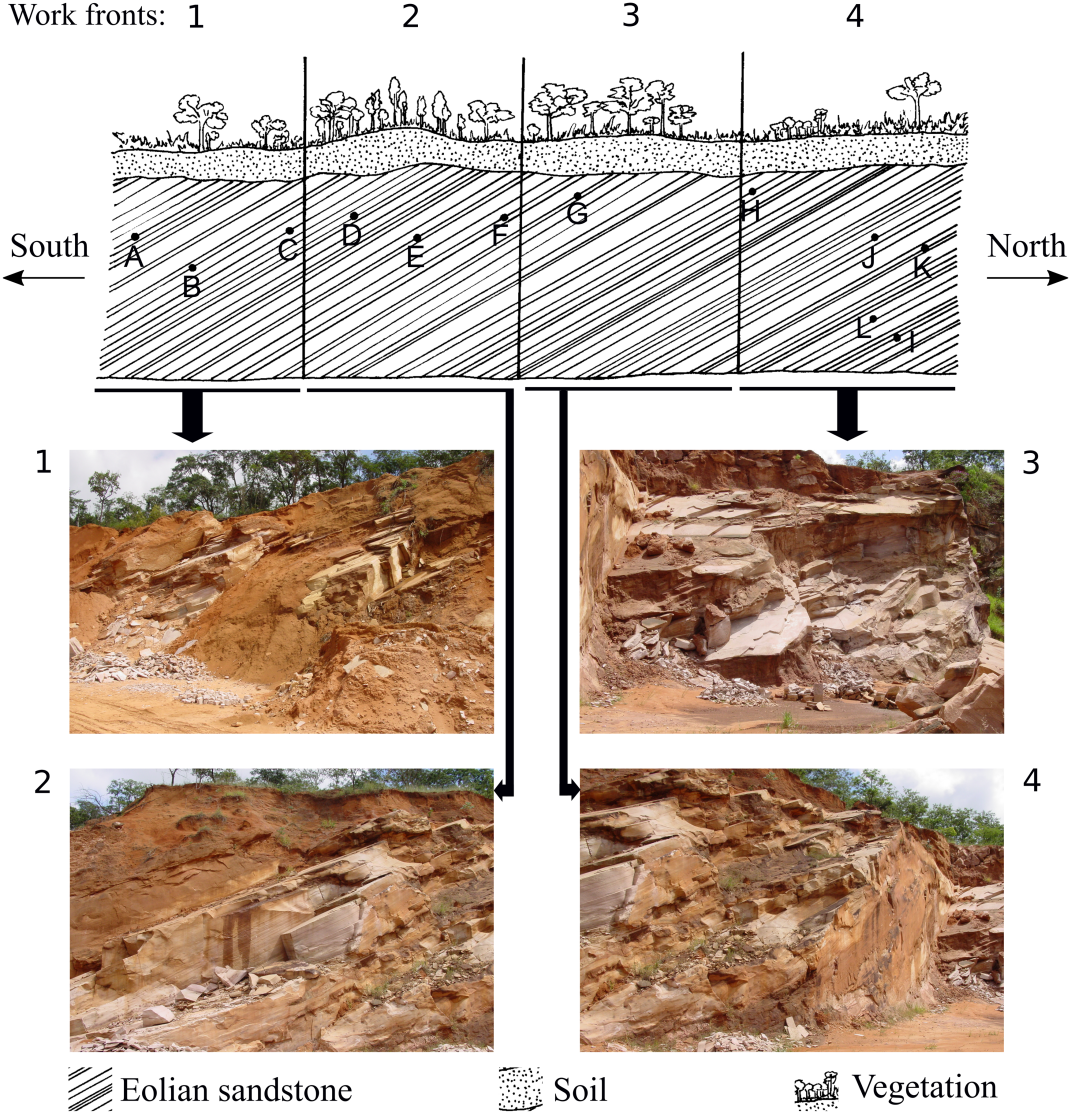
Representative drawing of the location of the work fronts of São Bento quarry. Photographs 1, 2, 3 and 4 are the work fronts rock outcrop in March 2004, during the commercial exploitation of the Botucatu sandstone and collection of all ichnofossils here described. (A), (B), (C), (D), (E), (F), (G), (H), (I), (J), (K), (L) are the relative location of the sites of occurrence of ichnofossils, as described by Fernandes (2005). The invertebrate ichnofossils occur in sites A, C, and D, in the work front 1. At present, the quarry is inactive, and the outcrop looks different because of the further exploitation and weathering. Drawing is not in scale (modified from Fernandes, 2005). The full-size image is in the Supplemental Information.

The Botucatu Formation is exposed in the Brazilian states of Mato Grosso, Mato Grosso do Sul, Goiás, Minas Gerais, São Paulo, Paraná, Santa Catarina, and Rio Grande do Sul with the same sedimentary system extending into Argentina, Uruguay, Paraguay, Namibia and South Africa, covering an area over 1.5 × 10^6^ km^2^ (Scherer & Goldberg, 2007). In São Paulo State, the Botucatu Formation outcrops as a northeast-southwest strip (Fig. 1), with monotonous deposits mostly consisting of yellowish to reddish, very fine- to coarse-grained sandstone, mainly quartz arenite and subordinately subarkose. The quartz arenite is texturally and mineralogically supermature, whereas the subarkose is texturally submature to mature and mineralogically mature (Wu & Caetano-chang, 1992). The consensus is that the Botucatu Formation represents a giant dry eolian depositional system (*erg*) based on the presence of large to medium-sized cross-stratified sandstones (Scherer & Goldberg, 2007) (Fig. 2), and on the basis of the mineralogical and textural maturity of the dominant deposits (Wu & Caetano-chang, 1992). The landscape was dominated by linear, crescentic and some star dunes, representing a hyperarid system, according the classification framework of Mountney (2004), with winds predominantly coming from the north in the northern part of the Paraná Basin (Scherer & Goldberg, 2007) where the study area is located (Araraquara City).

**Figure 3:**
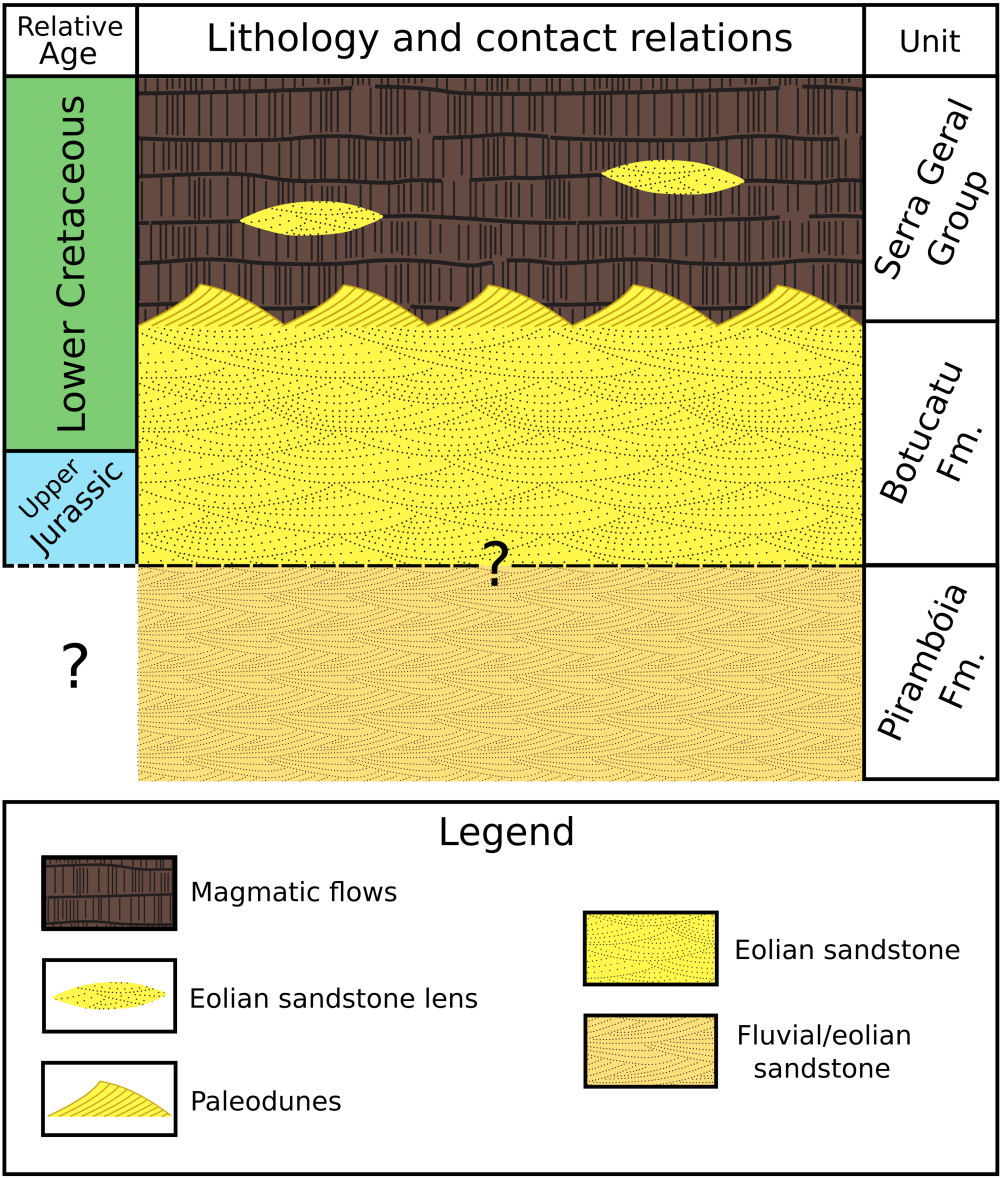
Simplified stratigraphic column showing the lithology, relative age and contact relationships between the Pirambóia, Botucatu and Serra Geral stratigraphic units. Not in scale.

Fluvial/eolian sandstone of the Pirambóia Formation occurs below the Botucatu Formation in the northern portion of the basin (State of São Paulo) (Milani et al., 2007, p. 287; Soares, Soares & Holz, 2008a, their fig2). The contact between these two formations is still controversial (Giannini et al., 2004, p. 282; Soares, Soares & Holz, 2008b, their fig. 2; p.126) (Fig. 3). The Botucatu Formation is overlain by the magmatic extrusive rocks of the Serra Geral Group (former Serra Geral Formation) (Milani et al., 2007; Fernandes et al., 2018) (Fig. 3). Lenses of eolian sandstone (paleodunes) in the Serra Geral Group indicate that the eolian depositional system was active during volcanism. The Botucatu Formation and the Serra Geral Group have a concordant contact because the flow of lava over the unconsolidated sand of the paleodunes created marks on the paleodune surfaces (e.g., striations, crescentic ridges), formed breccias (peperites), and also preserved the relief of the ancient dunes (Milani et al., 1998; Scherer, 2000; Scherer, 2002; Waichel et al., 2007; Holz, Soares & Soares, 2008; Waichel, Scherer & Frank, 2008).

U–Pb baddeleyite/zircon dating for the lowest sub-unit of the Serra Geral Group that makes concordant contact with Botucatu Formation in São Paulo State (Chapecó-type dacites) yields an age of approximately 134 Ma (Janasi, Freitas & Heaman, 2011). This radiometric date, together with the oldest paleomagnetic date of the Botucatu Formation from southern Brazil (Tamrat & Ernesto, 2006) indicates a Late Jurassic to Early Cretaceous age for this unit.

## Materials & Methods

### Paleontological material analyzed

The ichnofossils described here were collected between 1997 and 2005, at São Bento quarry (21°49′07.6″S48°04′28.8″W), in the municipality of Araraquara (São Paulo State). All the ichnofossils here described were found during the commercial exploitation of the successive layers of sandstone of the slipface of a single paleodune (that was more than 100 m long and 20 m high) of the Botucatu Formation (Fig. 2. The full-size figures are in Figs. S1 and S2). The paleodune slipface dips at 29° in the S-SW direction.

The São Bento quarry is currently inactive and is part of the Ouro Ichnofossiliferous Site, in Araraquara (Leonardi et al., 2002), a region with several abandoned ichnofossiliferous quarries. The sandstone slabs containing the ichnofossils here analyzed were collected from sites A, C and D (*sensu* Fernandes, 2005) of the São Bento quarry and are: LPP-IC-0028, LPP-IC-0029, LPP-IC-0030, LPP-IC-0031, LPP-IC-0032, LPP-IC-0033, LPP-IC-0034, LPP-IC-0035. Sandstone extraction at the São Bento quarry was done without scientific monitoring, and most of the fossils were in slabs ready to be cut, or already cut for commercialization. Therefore, no data is available regarding orientation of specimens with respect to the slopes. All of these slabs are deposited in the Paleoichnology Collection of the Laboratório de Paleoicnologia e Paleoecologia (LPP) of the Federal University of São Carlos (UFSCar), São Carlos campus.

The electronic version of this article in Portable Document Format (PDF) will represent a published work according to the International Commission on Zoological Nomenclature (ICZN), and hence the new names contained in the electronic version are effectively published under that Code from the electronic edition alone. This published work and the nomenclatural acts it contains have been registered in ZooBank, the online registration system for the ICZN. The ZooBank LSIDs (Life Science Identifiers) can be resolved and the associated information viewed through any standard web browser by appending the LSID to the prefix http://zoobank.org/. The LSID for this publication is: urn:lsid:zoobank.org:pub:53C73174-4645-40E1-BB7B-75856AEAEAF5. The LSID for the here described *Paleohelcura araraquarensis* isp. nov. is: urn:lsid:zoobank.org:act:7D4303AE-BB63-4474-B79C-AD39AB144917. The online version of this work is archived and available from the following digital repositories: PeerJ, PubMed Central and CLOCKSS

### Trackway measurements

**Figure 4:**
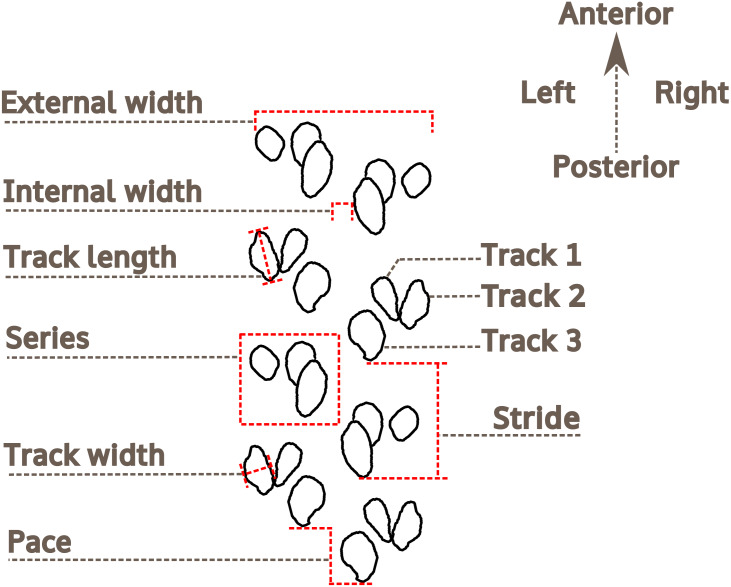
Nomenclature and measurements used for the analysis of *Paleohelcura araraquarensis* isp. nov. Measurements of the trackway and track characteristics.

The methodology and terminology of Trewin (1994), Braddy (2001) and Minter, Braddy & Davis (2007) for arthropod trackway description have been adopted herein (Fig. 4). For the description of preservation, the classification proposed by Seilacher (1964) is followed. For measurements of *Paleohelcura araraquarensis* isp. nov., four series on each side of the trackway were selected on slabs LPP-IC-0028, LPP-IC-0029, LPP-IC-0032, LPP-IC-0035. For those slabs with more continuous trackways, eight series on slab LPP-IC-0030 and nine series on slab LPP-IC-0031 were measured. The measured series are indicated in the photographs in Figs. S3 and S4. The series measured were chosen on the basis of the quantity and quality of the tracks on either side of the trackway. We attempted to select the series to be measured with regular distances between them along the trackway. In the slabs that show part and counterpart (LPP-IC-0029 with LPP-IC-0030 and LPP-IC-0031 with LPP-IC-0032), the same series were measured in the two slabs (i.e., negative epirelief and positive hyporelief). LPP-IC-0029 and LPP-IC-0032 present shorter trackways than their counterparts; therefore, only partial measurements were obtained. Measurements of internal and external width were taken; as were the pace, stride, and lengths and widths of the individual tracks (Fig. 4).

Trewin (1994, p. 813) proposed using the ratio between the external width and the internal width as a trackway parameter; however, such a relationship is not practical in the case of *Paleohelcura araraquarensis* isp. nov. since there are sections of the trackways where the internal width is zero, producing fractions with zero as divisor, and it is impossible to divide by zero (Kaplan, 2000). Therefore, we adopt the inverse of the relation proposed by Trewin (1994, p. 813), that is, internal width/external width (I/E), and we propose this as a standard for the description of trackways. The ratio between the length and width (L/W) of the tracks was also used to see if the tracks are circular (values close to one), or elliptical/elongated (values greater than one). The measurements were taken using a Vernier caliper. All measurements are listed in Table S1. The line drawing illustration of the holotype of *Paleohelcura araraquarensis* isp. nov. was produced using the Inkscape vector drawing program 0.92, whose license is free and open source (General Public License 3), using a photograph as a model for the footprint contour. All the graphic elements were produced with the aforementioned program.

## Results and Discussion

**Ichnotaxonomy.**

**Ichnogenus:** *Paleohelcura* Gilmore, 1926

**Type Ichnospecies:** *Paleohelcura tridactyla* Gilmore, 1926.

**Emended Diagnosis:** Trackways with external width greater than 20 mm, comprising two parallel track rows with series of commonly three tracks, but there can be fewer or up to four tracks per series. Series have alternating to staggered symmetry. Tracks vary from slightly elliptical to tapered or circular and can be in a linear or triangular arrangement within series. A medial impression may be present.

**Remarks:** Historically, many arthropod trackway ichnotaxa were inadequately described and illustrated, at times based on few and/or poorly preserved specimens (Trewin, 1994, p. 821). Several trackway ichnotaxa with series of at most four tracks have been described, resulting in potential junior synonyms. Analysis of trackways from the Permian of Germany and southwestern United States (Minter, Braddy & Voigt, 2007; Minter & Braddy, 2009) showed intergradations between several ichnotaxa, underscoring morphological and preservational variations, due to small variations in locomotion or characteristics of the substrate.

*Paleohelcura* (Gilmore, 1926) was originally described from the lower Permian Coconino Sandstone of western United States and subsequently described in other studies dealing with the ichnology of this and other units (Toepelman & Rodeck, 1936; Brady, 1939; Brady, 1947; Brady, 1961; Alf, 1968; Sadler, 1993; Braddy, 1995; Lucas & Lerner, 2004; Morrissey & Braddy, 2004; Voigt, Small & Sanders, 2005; Batchelor & Garton, 2013; Stoller, Rowland & Jackson, 2013). Only *Paleohelcura tridactyla* is accepted as a valid ichnospecies, and four forms are regarded as junior synonyms: *P. dunbari* (Brady, 1961), *P. delicatula* (Fischer, 1978), *P. badensis* Kozur, Loffler & Sittig (1994), and *P.? lyonsensis* (Toepelman & Rodeck, 1936), which was provisionally included in *Paleohelcura* when described.

Sadler (1993) noted intergradations between *P. tridactyla* and *P. dunbari,* but retained them as separate ichnospecies because she considered the two ichnotaxa as morphologically different. On the contrary, it has been argued that the morphologic differences between *P. tridactyla* and *P. dunbari* are minor, with intergradations between the two, and so *P. dunbari* should be regarded as a junior synonym of *P. tridactyla* (Minter, Braddy & Davis, 2007; Minter & Braddy, 2009). *Paleohelcura delicatula* is only known from a single specimen that consists of comma-shaped tracks, opposite symmetry, and small size compared with *P. tridactyla* (Fischer, 1978). New ichnotaxa should not ideally be erected on the basis of single specimens and the characteristics presented are not reliable to erect a new ichnospecies.

Therefore, we regard *Paleohelcura delicatula* as a junior synonym of *Stiaria intermedia*. *Paleohelcura badensis* is regarded as a junior synonym of *Stiaria intermedia* (Minter & Braddy, 2009). *Paleohelcura? lyonsensis* has been considered a junior synonym of *P. tridactyla* (Braddy, 1995, p. 221). Gilmore (1927) erected *Triavestigia niningeri,* and Kozur, Loffler & Sittig (1994) referred it to *Paleohelcura* as a distinct ichnospecies; however, the holotype consists of an incomplete trackway, and the arrangement of the tracks within a series suggests that it is a junior synonym of *P. tridactyla* (Braddy, 1995, p. 221; Minter & Braddy, 2009).

*Paleohelcura tridactyla* is similar to *Stiaria intermedia* Smith, 1909 (Walker, 1985; Minter & Braddy, 2009). *Stiaria* was described from continental fine-grained sediment lenses within andesites (Walker, 1985; Phillips & Smith, 2008, p. 5) from the Lower Devonian Old Red Sandstone of Scotland, and was revised by Pollard & Walker (1984), and Walker (1985), with the latter paper erecting a neotype, lectotype and paratypes not previously assigned to this ichnotaxon. Both ichnogenera may possess a medial impression and linear series with two to four tracks (Brady, 1947; Walker, 1985; Minter & Braddy, 2009). In fact, Walker (1985) suggested that *Paleohelcura* should be regarded, at least in part, as a junior synonym of *Stiaria.*

*Stiaria quadripedia* is a similar ichnospecies and was also revised by Walker (1985), but differs from *Paleohelcura* by presenting bifid or trifid tracks, which are possible to delineate in finer-grained sediments. *Stiaria intermedia* consists of trackways with up to three circular tracks, similar to *Paleohelcura*. In contrast, *Stiaria quadripedia* may have four tracks, and is larger than *S. intermedia.* Size is not regarded as an appropriate ichnotaxobase (Bertling et al., 2006), but analysis of the external widths of specimens assigned to *Stiaria intermedia* and to *Paleohelcura tridactyla* has shown that they fall into two separate size classes (Minter & Braddy, 2009). Whilst not separated by an order of magnitude, a working model was proposed (Minter & Braddy, 2009) whereby *Stiaria intermedia* should be used for trackways with an external width of less than 20 mm, and *Paleohelcura* for those with an external width greater than 20 mm, like the trackways here described (see Table 2).

**Table 2:** Arithmetic mean of the measurements of the trackway. (I/E) Internal Width/External Width. LPP-IC-0033 and LPP-IC-0034 were not measured because of their poor preservation.

Specimen	External width mean (mm)	Internal width mean (mm)	I/E Mean	Pace mean (mm)	Stride mean (mm)
LPP-IC-0028	22,80	2,43	0,11	12,73	12,80
LPP-IC-0029	23,43	2,70	0,11	10,78	11,30
LPP-IC-0030	22,75	1,68	0,07	5,49	10,92
LPP-IC-0031	23,22	1,41	0,06	6,13	11,75
LPP-IC-0032	22,10	1,95	0,09	10,90	11,40
LPP-IC-0035	23,55	3,11	0,10	11,18	11,27
Mean (mm)	22,97	2,21	0,09	9,53	11,57
Standard deviation/Mean	0,05	0,57	0,62	0,14	0,11

The clarification of the ichnotaxonomic status of *Stiaria intermedia* and *Paleohelcura tridactyla* remains to be achieved through examination of their holotypes and neotypes (Minter & Braddy, 2009). In any case, *Paleohelcura* is a well-accepted ichnotaxon, which has been recorded extensively. The working model proposed by Minter & Braddy (2009) has been adopted in many papers (Lucas et al., 2005; Minter & Braddy, 2009; Fillmore, Lucas & Simpson, 2010; Poschmann & Braddy, 2010; Batchelor & Garton, 2013; Getty et al., 2013; Getty et al., 2017; Bernardi, Marchetti & Gobbi, 2018; Uchman, Gazdzicki & Blazejowski, 2018), and is endorsed here. *Paleohelcura araraquarensis* isp. nov. is placed in *Paleohelcura* instead of *Stiaria* because it exhibits an external width greater than 20 mm.

Paleohelcura araraquarensis isp. nov.

Figures 5 and 6A.

**Figure 5:**
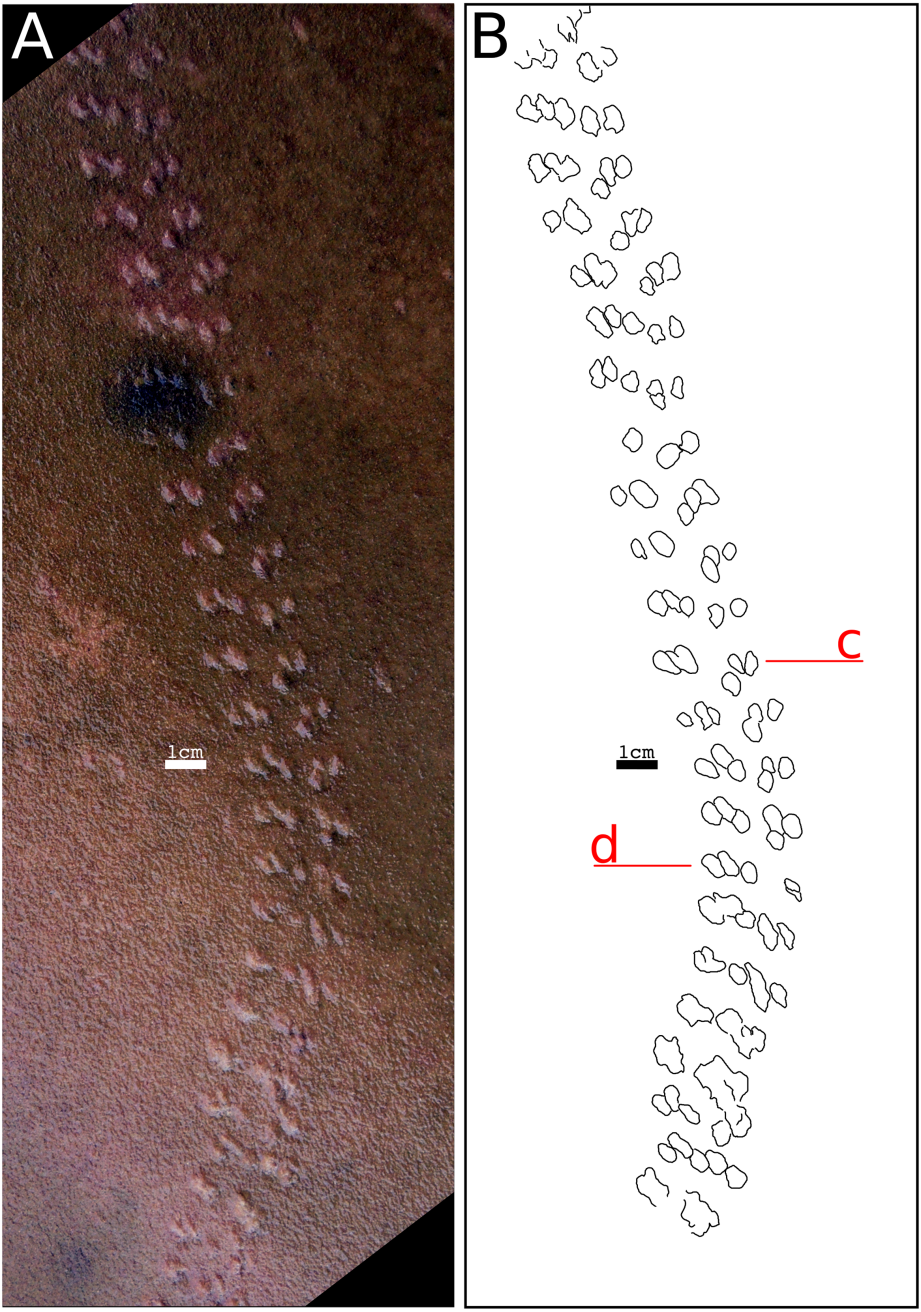
Holotype of *Paleohelcura araraquarensis.* isp. nov. (LPP-IC-0028). (A) Photograph of LPP-IC-0028 slab showing the positive hyporelief of *Paleohelcura araraquarensis* isp. nov. (B) Representative scheme of the holotype of *Paleohelcura araraquarensis* isp. nov. Lowercase letters c and d indicate examples of footprint orientation within the series. The producer walked from bottom to top.

**Figure 6:**
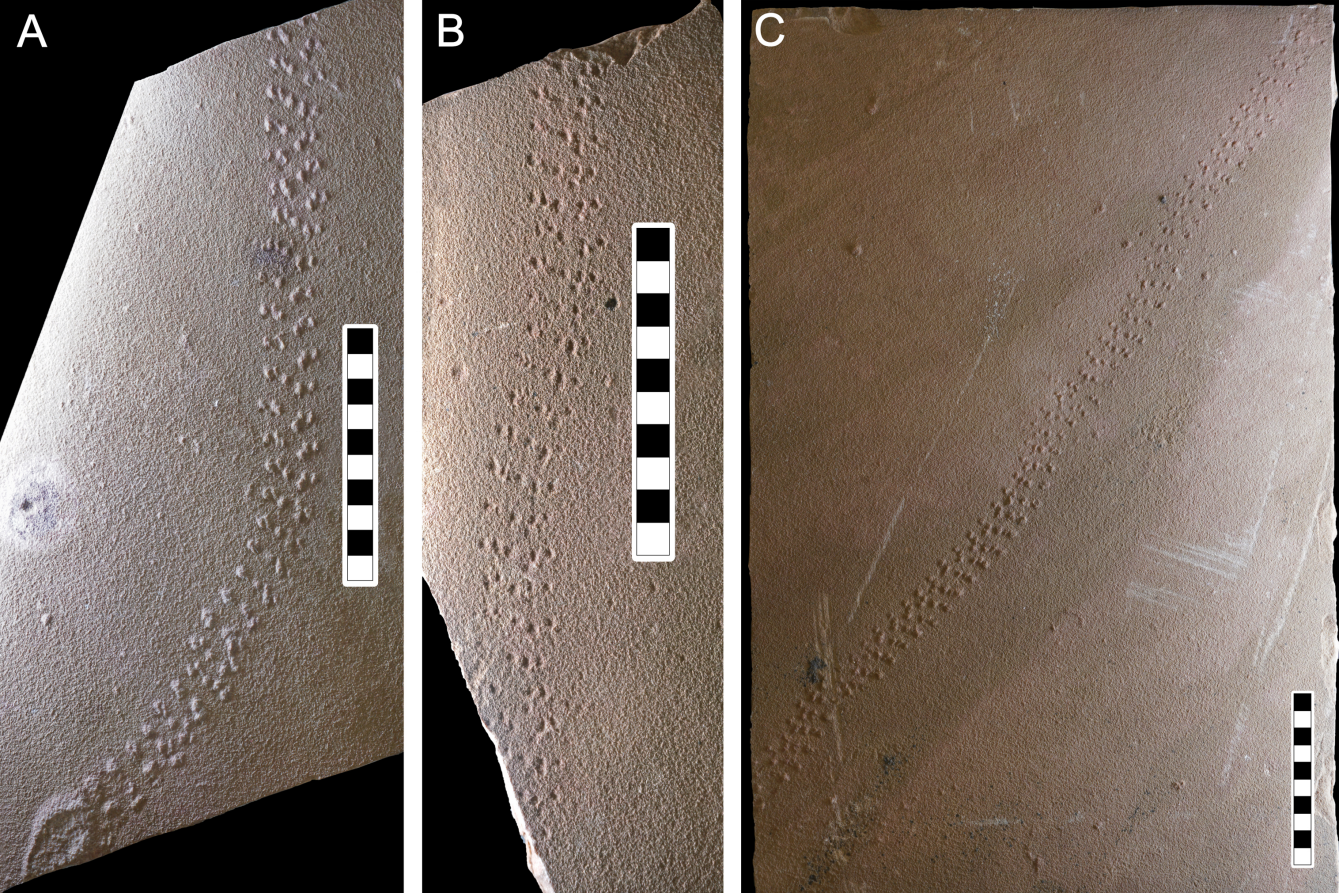
Photographs of some of the slabs bearing *Paleohelcura araraquarensis* isp. nov. (A) LPP-IC-0028 (holotype). (B) LPP-IC-0029 and (C) LPP-IC-0030. The producer walked from bottom to top. Scale bar: 10 cm.

**Horizon and type locality:** São Bento Group, Botucatu Formation; Locality: Ouro; municipality: Araraquara; São Paulo State (Fig. 1); São Bento quarry (Corpedras company) (Fig. 2), geographical coordinates: 21°49′07.6″S 48°04′28.8″W, altitude: 670 m.

**Holotype:** LPP-IC-0028: sandstone slab showing slightly curved trackway, preserved in positive hyporelief over a length of 34 cm. Reposited in the Paleoichnology collection of the Laboratório de Paleoecologia e Paleoicnologia (LPP) of the Federal University of São Carlos (UFSCar) *campus* São Carlos-SP.

**Paratypes:** Sandstone slabs: LPP-IC-0029 (negative epirelief) and its counterpart LPP-IC-0030 (positive hyporelief); LPP-IC-0031 (negative epirelief) and its counterpart LPP-IC-0032 (positive hyporelief); LPP-IC-0033 (negative epirelief) and its counterpart LPP-IC-0034 (positive hyporelief); LPP-IC-0035 (negative epirelief) and with no apparent counterpart slab. All slabs are reposited in the Paleoichnology collection of the Laboratório de Paleoecologia e Paleoicnologia (LPP) of the Federal University of São Carlos (UFSCar) *campus* São Carlos-SP.

**Etymology:** It is dedicated to the city of Araraquara, São Paulo State, where these trackways were found, along with most of the ichnofossils of the Botucatu Formation.

**Diagnosis:** Trackways composed of two rows, whose internal width between the rows is less than one-quarter of the external width; with alternating to staggered series, consisting of up to three tracks with different sizes that may vary from slightly elongated to tapered or circular in shape.

**Figure 7:**
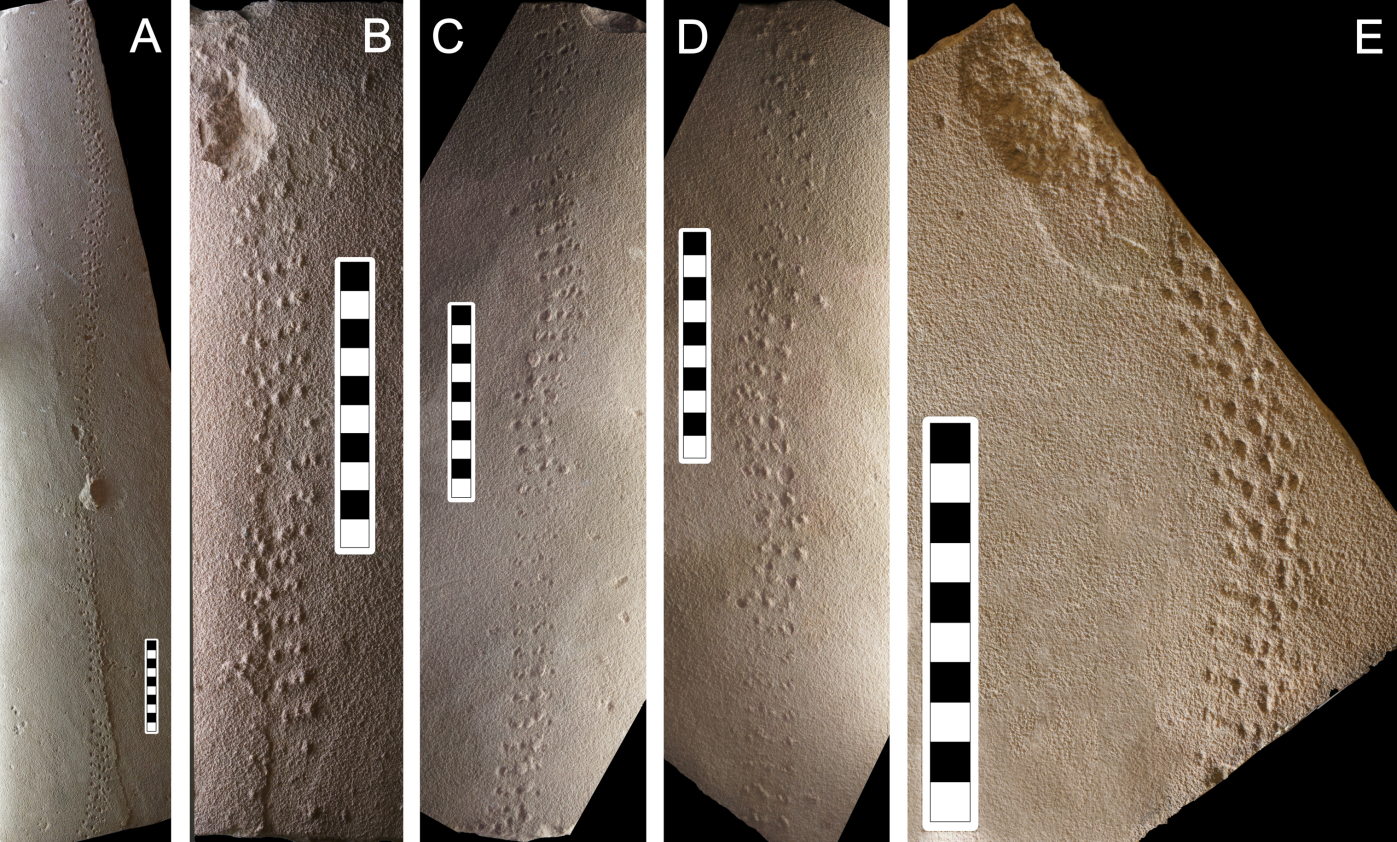
Photographs of some of the slabs bearing *Paleohelcura araraquarensis* isp. nov. (A) LPP-IC-0031. (B) LPP-IC-0032. (C) LPP-IC-0033. (D) LPP-IC-0034. (E) LPP-IC-0035. The producer walked from bottom to top. Scale bar: 10 cm.

**Description:** Due to the similarity between the size of the sandstone grains and the size of the locomotory appendages of the producer, the tracks of *Paleohelcura araraquarensis* isp. nov. in all the analyzed slabs have little definition. The following slabs have counterparts: LPP-IC-0029 (negative epirelief) and LPP-IC-0030 (positive hyporelief), Figs. 6B and 6C, respectively; LPP-IC-0031 (negative epirelief) and LPP-IC-0032 (positive hyporelief), Figs. 7A and 7B, respectively; LPP-IC-0033 (negative epirelief) and LPP-IC-0034 (positive hyporelief), Figs. 7C and 7D, respectively. The organization of the tracks in the series follows a pattern with two, usually smaller, tracks grouped anteriorly and often more externally, and a longer track more posteriorly and commonly internally positioned (Fig. 5C). In places, the series adopt a linear configuration (Fig. 5D) that, despite showing some recurrence, is not an appropriate feature for the diagnosis because it is a variation in the more consistent triangular pattern shown in Fig. 6C.

The arithmetic means of the measurements of the trackway parameters are summarized in Tables 2 and 3. All measurements are in the Table S1. The average external width of the trackways is 22.97 mm, and the average internal width is 2.21 mm, with absolute values of the latter varying from 3.80 mm to 0 mm (i.e., no internal separation between series). The internal trackway width is, on average, approximately one-tenth of the outer trackway width. The ratio between the length and width of the tracks (L/W) is always greater than one, which indicates that they are elongated. It is rare in *Paleohelcura araraquarensis* isp. nov. for tracks to have lengths and widths with similar values, that is, with a circular shape.

**Table 3:** Arithmetic mean of the measurements of each track in the series. (L/W) Length /Width. LPP-IC-0033 and LPP-IC-0034 were not measured because of their poor preservation.

Specimen	Track 1 (Mean)			Track 2 (Mean)			Track 3 (Mean)		
	Length (mm)	Width (mm)	L/W	Length (mm)	Width (mm)	L/W	Length (mm)	Width (mm)	L/W
LPP-IC-0028	5,41	3,14	1,73	4,94	3,03	1,63	5,11	3,79	1,35
LPP-IC-0029	4,98	2,83	1,76	4,38	3,41	1,28	4,28	3,37	1,27
LPP-IC-0030	4,43	2,73	1,62	4,13	2,77	1,49	4,32	3,21	1,35
LPP-IC-0031	4,60	2,97	1,55	4,53	3,07	1,47	4,81	3,77	1,27
LPP-IC-0032	4,96	3,10	1,60	4,44	3,01	1,47	4,77	3,53	1,35
LPP-IC-0035	4,28	2,84	1,51	4,73	3,28	1,44	5,41	3,74	1,45
Mean (mm)	4,78	2,93	1,63	4,52	3,09	1,47	4,78	3,57	1,34
Standard deviation/Mean	0,19	0,17		0,17	0,15		0,15	0,15	

There were variations observed between track measurements in the negative epirelief and their corresponding counterpart slab. This may have two causes: (i) subjectivity may have caused variation in the measurement of tracks of different toponomy, one in negative epirelief and another one in positive hyporelief (i.e., methodological bias); or (ii) it would suggest that arthropod tracks are susceptible to another type of alteration, after the production of the footprint and the lithification of the substrate, generated by the splitting of the layers in to part and counterpart slabs (i.e., taphonomic bias). In this situation, tracks can lose parts, become smaller, or retain the sediment of the counterpart slab, thereby modifying their size. As such, the measurements taken from an ichnofossil may not correspond precisely to the size of the tracks left by the animal when the substrate was unconsolidated. It was not possible to take accurate track measurements of the counterpart slabs LPP-IC-0033 and LPP-IC-0034 (Figs. 7C and 7D) due to the poor preservation of the tracks, but they are included as paratypes because they represent part of the variation that *Paleohelcura araraquarensis* isp. nov. can exhibit, whether due to preservation, taphonomic process or produced by the disaggregation of the layers. The paratypes (LPP-IC-0029, LPP-IC-0030, LPP-IC-0031, LPP-IC-0032, LPP-IC-0033, LPP-IC-0034) did not present significant differences in relation to the holotype (LPP-IC-0028).

**Figure 8:**
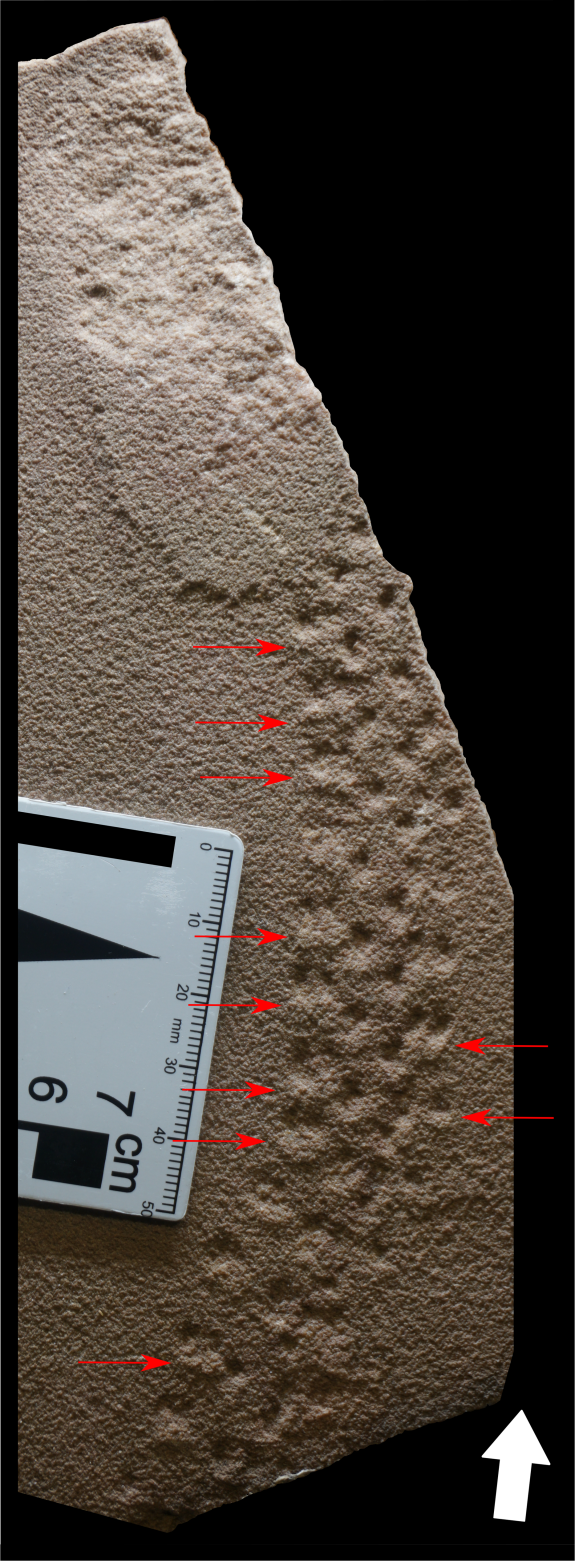
Specimen LPP-IC-0035 of *Paleohelcura araraquarensis* isp. nov. in negative epirelief exhibiting deformation in the sediment by the effort of locomotion of the animal. Red arrows: strain by locomotion effort; White arrow: the direction of movement of the animal. The light source is at the top of the photo.

Specimens in LPP-IC-0028, LPP-IC-0029, LPP-IC-0030, LPP-IC-0031, LPP-IC-0032, LPP-IC-0035 show sediment displacement associated with the tracks (Fig. 8). In slabs with positive hyporelief (LPP-IC-0028, LPP-IC-0030, LPP-IC-0032), the displacement appears as a faint depression attached to the track. This displacement indicates the direction of movement, being located on the opposite side from the direction of movement of the animal, generated by the effort that the locomotory appendage applied to the unconsolidated substrate to generate propulsion. However, we cannot rule out the possibility that the displacement could have been generated by sliding of the animal caused by the slope of the dune; this interpretation is less likely because the orientation of the displacement is the same in all the specimens that exhibit it. Therefore, we consider that the displacement was more likely to have been generated by the propulsion of the animal over the sand.

The commercial extraction of the sandstone at the São Bento quarry was undertaken without scientific monitoring, and most of the fossils were in slabs ready to be cut, or already cut for commercialization. Thus, the fossils were rescued and there was no record of the orientation of the slabs in relation to the slope or whether the holotype and the paratypes could be parts of the same large but fragmented individual trackway.

### Comparisons

The main difference between *Paleohelcura araraquarensis* isp. nov. and *Paleohelcura tridactyla,* and its junior synonyms (revised in Remarks section of the ichnogenus *Paleohelcura*) is the ratio of the internal width to the external width. *Paleohelcura araraquarensis* isp. nov. has an internal width equal to or less than one-quarter of the external width (on average one-tenth of the external width). The internal width in other ichnospecies of *Paleohelcura* is greater than one-quarter of the external width, even in *Paleohelcura* with narrow internal widths like some of from the lower Permian Robledo Mountains and Coconino Sandstone of the USA that have external widths of similar or lower absolute values to *P. araraquarensis* isp. nov. (Minter & Braddy, 2009, their fig. 30; 31), and those from the Permian Lyons Sandstone of Colorado (USA) (Toepelman & Rodeck, 1936, their fig. 1).

The triangular series arrangement and ellipsoidal tracks shown by *Paleohelcura araraquarensis* isp. nov. (Fig. 5C) slightly resemble *Lithographus* and some of its junior synonyms. *Lithographus* comprises trackways with series of up to three tracks with alternate to staggered symmetry but differs from *Paleohelcura* in that the tracks are linear and have varied orientations with respect to the midline of the trackway (Minter & Braddy, 2009). Several ichnogenera were synonymized with *Lithographus* by Minter & Braddy (2009). Series within *Lithographus* and *P. araraquarensis* isp. nov. share two usually smaller tracks that are grouped anteriorly and commonly more externally, and a longer track that is positioned more posteriorly and internally. We suggest that this characteristic may reflect the pterygote insect leg arrangement (see Trace-fossil producer section).

The main morphological characteristic of *Lithographus* are its linear tracks with varied orientations, which differs from the rounded to elliptical tracks of *Paleohelcura araraquarensis* isp. nov., whose long-axes are subparallel to the midline of the trackway. It is, therefore, more reasonable to assign the trackways described here to *Paleohelcura*, and to establish a new ichnospecies for forms with a narrow internal width.

### Trace-fossil producer

**Figure 9:**
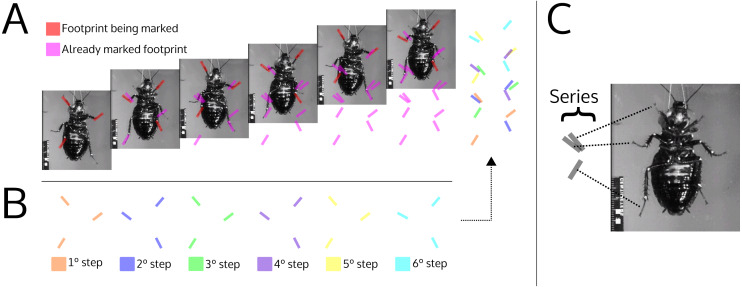
Illustration simulating the marks left by a cockroach while walking (ventral view). (A) The photographs show the advancement of a cockroach and the marks that would be left by its feet. The red footprints on the photograph indicate footprints that have just been produced; magenta footprints indicate already produced footprints. (B) Footprints produced in each step, broken down by color. (C) Association between the footprints within the series and the pairs of feet that produced them. The photographs are frames of a video courtesy of R.E. Ritzmann showing a cockroach (*Blaberus discoidalis*) in ventral view walking on an oiled glass plate (Video S1).

Although series arrangement and track shape are variable, the most common pattern identified is useful for making neoichnological comparisons. An alternating tripod gait is a relatively robust locomotion pattern for Hexapoda (Wöhrl, Reinhardt & Blickhan, 2017a). Arachnids, although possessing four pairs of locomotory appendages, can produce series with three tracks, either by adopting a hexapedal gait or for taphonomic reasons (Davis, Minter & Braddy, 2007; Schmerge, Riese & Hasiotis, 2013). Trackways comprising series with alternating symmetry on either side of the medial line, and with a maximum of three tracks per series, indicate that the animal maintained at least three feet on the ground while walking (Fig. 9). Therefore, we restrict the discussion of the producer to the Arachnida and Hexapoda.

**Figure 10:**
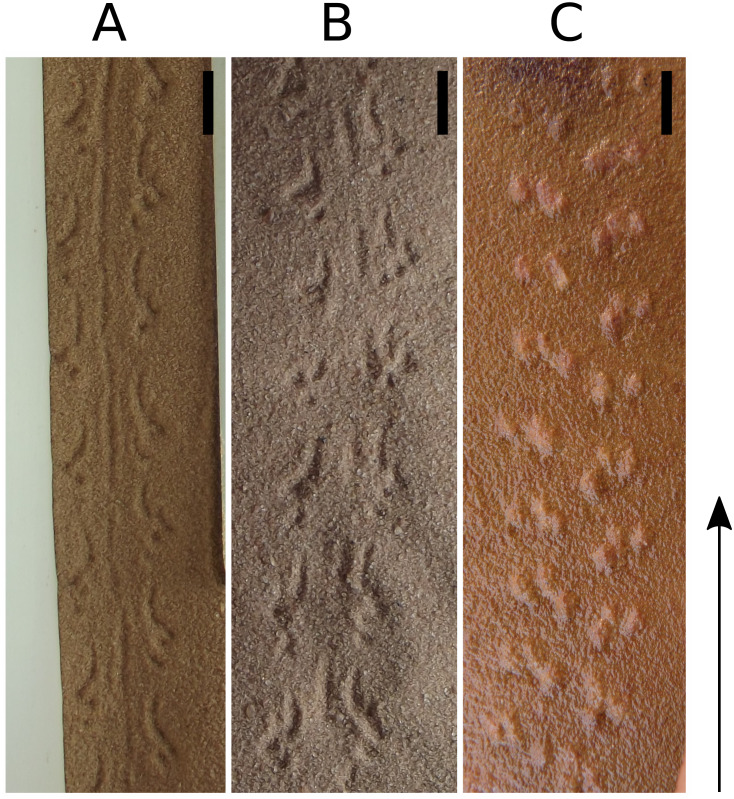
Scorpion *Tityus serrulatus.* tracks in sand (A and B) and *Paleohelcura araraquarensis* isp. nov. holotype LPP-IC-0028 (C). Scale bar: 1 cm. The arrow indicates the direction of the animal movement. (A) and (B) photographs courtesy of Ravi Sampaio, 2015.

The trackways made by spiders do not resemble *Paleohelcura araraquarensis* isp. nov. because they have larger internal width, circular tracks, and a different series arrangement Davis, Minter & Braddy, 2007, their Fig. 9). When scorpions leave series with three tracks they resemble *P. araraquarensis* isp. nov., but with a different arrangement of tracks within the series. In *P. araraquarensis* isp. nov., there are two tracks anteriorly positioned, and a usually longer track more posteriorly and internally positioned. In scorpion trackways, there are two tracks more posteriorly positioned and one track that lays anteriorly, and the former are usually longer and commonly most internally positioned (Fig. 10; Davis, Minter & Braddy, 2007, their fig. 7–8). In addition, even small scorpions, with comparable size to the animal that made *Paleohelcura araraquarensis* isp. nov., leave trackways with a large internal width, differing from the narrow internal width of *P. araraquarensis* isp. nov. (Fig. 10). Modern scorpions are very similar to Paleozoic scorpions (Polis, 1990, p. 2); therefore, neoichnological studies provide strong grounds to exclude scorpions as producers of *P. araraquarensis* isp. nov.

Neoichnological studies with cockroaches produced trackways with elongated tracks because they walk on tarsal segments (tarsomeres) (Davis, Minter & Braddy, 2007). Therefore, segmented tarsi appear to be an important feature to generate tracks similar to those of the ichnogenus *Lithographus* and its junior synonyms. Within the Hexapoda, Protura, Diplura, Monura and Collembola possess undivided tarsi (Kristensen, 1998, p. 289; Bitsch & Bitsch, 2000, p. 140), probably producing tracks similar to those of an arachnid because they also do not have segmented tarsi. Only the true insects (Zygentoma, Archaeognatha, and Pterygota) have segmented tarsi (Kristensen, 1998, p. 289; Bitsch & Bitsch, 2000; Gorb & Beutel, 2001, p. 534). The neoichnology of representatives of Zygentoma and Archaeognatha revealed that they produced circular/elliptical to elongated tracks (Getty et al., 2013), which resemble those produced by arachnids. In this case, it is due to the low mobility of the tarsomeres, which lead to the digitigrade posture (on the pretarsus). The increased mobility of the tarsomeres in Pterygota is linked to the evolutionary pressure to climb and walk on a variety of new substrates due to their ability to fly and the necessity to hold onto leaves and plant stems (Gorb & Beutel, 2001, p. 533).

Most Pterygota walk on tarsomeres (Manton, 1972; Zollikofer, 1994, p. 98; Frazier et al., 1999; Boggess et al., 2004; Davis, Minter & Braddy, 2007; Gladun & Gorb, 2007; Clemente & Federle, 2008; Wöhrl, Reinhardt & Blickhan, 2017b); cockroach video in Video S1 of this publication, courtesy of R.E. Ritzmann). As observed in neoichnological experiments on cockroaches (Davis, Minter & Braddy, 2007), they all probably produce elongated or elliptical tracks. Nevertheless, it is not possible to assume this for all pterygote insects without more neoichnological experiments because there are species that walk on a few distal tarsomeres and on their pretarsus (Niederegger & Gorb, 2003; Gladun & Gorb, 2007; Endlein & Federle, 2015), thereby probably producing elongated tracks, but less so than those of full plantigrade insects.

**Figure 11:**
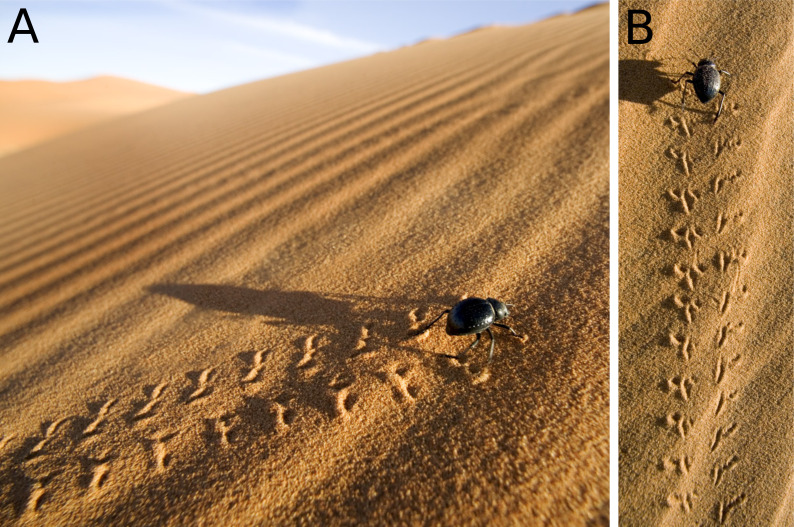
Darkling beetles (Tenebrionidae) and their tracks from Morocco dunes. (A) Oblique view of the trackway and the walking darkling beetle. (B) Perpendicular view of the trackway and the walking darkling beetle. The photos were not taken with scales, but these beetles are around 2 cm long. Photographs courtesy of Martin Harvey, 2004.

Trackways of some desert darkling beetles (Tenebrionidae) closely resemble *Paleohelcura araraquarensis* isp. nov. in that they comprise a narrow internal width, elliptical tracks, and series that usually exhibit the same arrangement of *P. araraquarensis* isp. nov. (Fig. 11). Despite being made by pterygote insects, they do not show strong linear tracks like the cockroaches used in experiments by Davis, Minter & Braddy (2007) that made trackways similar to *Lithographus*. Even with segmented tarsi, these Tenebrionidae leave elliptical tracks just like in *P. araraquarensis* isp. nov. This is probably due to the small size of the animal compared with the sand grain size, which diminishes the resolution of the tracks. The cockroaches used by Davis, Minter & Braddy (2007) were relatively large compared to the grain size of the substrate, creating trackways of greater than 40 mm external width, and so it is expected that the tracks reflected more faithfully the morphology of the locomotory appendages.

The series arrangement in the trackways of Tenebrionidae are also similar to those observed in *Paleohelcura araraquarensis* isp. nov. and cockroach trackways, with two usually smaller tracks grouped anteriorly and often more externally and a longer track more posteriorly and often internally positioned. This arrangement could be related to the positions of the legs and the role of each leg in the gait of pterygote insects. Therefore, we consider that the producer of *P. araraquarensis* isp. nov. would probably be an insect from the Pterygota rather than an arachnid, and suggest Recent Tenebrionidae as a plausible analog, noteworthy for their high abundance and diversity in deserts, as a result of their striking ability to adapt to hyperarid settings (Cloudsley-Thompson, 2001). This contrasts with the interpretations of the producers of other ichnospecies of *Paleohelcura*, inferred to have been made by scorpions and spiders (Davis, Minter & Braddy, 2007). Although a beetle affinity is proposed, more neoichnological studies are necessary to discriminate other potential producers since the diversity of pterygote insects is high (Clapham et al., 2016). In addition, it will enable greater understanding of the effect on trackway morphology of the interaction among the size of an arthropod, the morphology of its limbs, the grain size, and the moisture in the substrate.

### Paleoautoecologic implications

Looking at modern deserts, among Pterygota reaching similar size, the Coleoptera are one of the most conspicuous and abundant animals in arid environments (Holm & Scholtz, 1980; Cloudsley-Thompson, 2001; Whitford, 2002). Some, like Tenebrionidae, possess several morphological, physiological, and mainly behavioral adaptations to deal with extremely dry and hot environments (ultra-psammophilous) (Cloudsley-Thompson, 2001). Beyond their remarkable incidence in deserts, these animals play an ecological role in dune deserts of consuming detritus among sand grains and being able to rely on this biomass even when there is no primary production during dry periods in hyperarid deserts (Seely & Louw, 1980; Southgate, Masters & Seely, 1996).

The producer of *Paleohelcura araraquarensis* isp. nov. being a pterygote insect could predominately have been a herbivore, carnivore, or an omnivore. Omnivory is important in desert food webs since primary productivity is limited by moisture availability (Polis, 1991). Such an animal would be able to feed on the organic matter that accumulated in the paleodunes, but also be capable of adopting herbivorous, saprophagous, or carnivorous diets when appropriate. Tenebrionidae are a notable example of this in modern dune deserts (Koch, 1961; Holm & Scholtz, 1980; Robinson & Seely, 1980; Seely & Louw, 1980; Cloudsley-Thompson, 2001). If it had been carnivorous, the producer of *Paleohelcura araraquarensis* isp. nov. could be a predatory insect, like some species of Carabidae (Coleoptera) living in dunes of the Negev desert (Filser & Prasse, 2008).

Despite *Paleohelcura araraquarensis* isp. nov. sharing some morphological characteristics with trackways of some dune desert Tenebrionidae (see**** Trace-fossil producer section), it is not possible to establish a confident link between the fossil tracks and the group without eliminating other Pterygota as candidates through neoichnology or the discovery of associated body fossils. Nevertheless, the existence of *Taenidium* isp. and *Skolithos linearis* burrows (Fernandes, Netto & Carvalho de, 1988; Fernandes, Carvalho & Netto, 1990; Fernandes, 2005; Peixoto et al., 2016) shows that the Botucatu paleodesert played host to a community of omnivorous detritivorous insects just like modern dune deserts, where they comprise most of the animal biomass on the dune slip-face (e.g., Namib dune desert: Seely & Louw, 1980). *P. araraquarensis* isp. nov. could be produced by an insect of this community like omnivorous Tenebrionidae, which produces similar trackways in modern dune deserts.

### Paleoecology of the Botucatu desert

**Figure 12:**
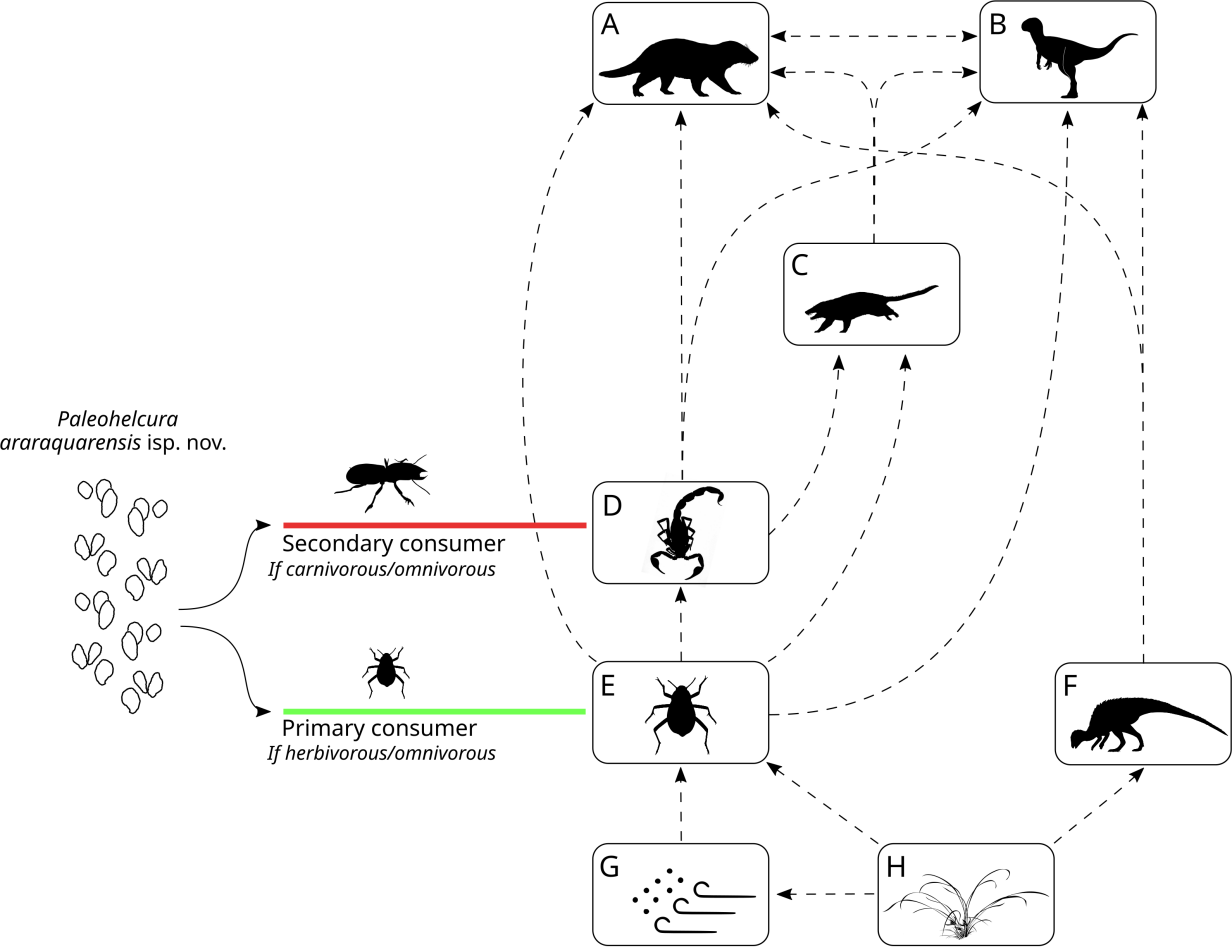
Reconstruction of the Botucatu paleodesert food web based on the interpretation of the probable producers of the ichnofossil of Botucatu Formation. The dashed arrows represent the ecological relations and the flow of energy in the Botucatu paleodesert. (A) Large mammaliaform. (B) Theropod dinosaurs. (C) Small mammaliaform. (D) Arachnids and possible insects. (E) Insects. (F) Ornithopod dinosaurs. (G) Detritus in the sand and blown by the wind. (H) Plants. On the left, the possible ecological roles that the *Paleohelcura araraquarensis* isp. nov. producer could have played in the Botucatu desert are shown. This scheme was made with modifications of artworks of different authors: Carivorous Insect from Rafael Pasini; Scorpion in (D) from Gareth Monger; (A) and (C) Mammaliaforms from Ceri Thomas; (B) Theropod dinosaur from Frederic Wierum; (F) Ornithopod dinosaur from Nobumichi Tamura. All artworks are under the CC-BY-SA 4.0 (https://creativecommons.org/licenses/by/4.0/).

The trace-fossil record of the Botucatu Formation may provide indirect evidence on the abundance and diversity of organisms that inhabited these eolian dunes. Through the interpretation of the phylogenetic affinity and probable nutrient source of the producers, it is possible to make broad inferences about the ecological relationships of these organisms in the context of the Late Jurassic—Early Cretaceous Botucatu desert. Modern desert food webs are complex due to a high frequency of omnivory, generating highly connected food webs, with species interacting with many predators and prey (Polis, 1991). Omnivorous insects comprise most of the animal biomass on the dune slip-face of modern deserts (e.g., Namib dune desert; Seely & Louw, 1980). A similar situation is envisaged for the Botucatu paleodesert. *Taenidium* isp. and *Skolithos linearis* were most likely produced by insects feeding on detritus among the sand grains (Figs. 12G to 12E), like Tenebrionidae in modern dune deserts. Those with compatible size could produce *Paleohelcura araraquarensis* isp. nov. when walking on the sand. Insects feeding on living plant material as omnivores or herbivores could also produce *P. araraquarensis* isp. nov. (Fig. 12H to 12E). Accordingly, relying on plant material, the detritivorous and herbivorous invertebrates are regarded as part of the primary consumers in the trophic web of the Botucatu paleodesert.

The geomorphological characteristics of an *erg* create habitats with abiotic characteristics that determine the productivity, biomass and diversity found in the interdune, and the windward and slip-face subenvironments of the dune field (Seely & Louw, 1980; Southgate, Masters & Seely, 1996). Even though the slip-face occupies a small area of the *erg*, when compared to the windward or interdune areas (in the case of *ergs* with well-spaced dunes), and is a region of low plant growth, it has a high concentration of biomass per unit area in the form of detritus, concentrated by the wind mainly at the base of the dunes (Seely & Louw, 1980; Southgate, Masters & Seely, 1996). This detritus originates from adjacent subenvironments, more conducive to plant growth where there is moisture (Seely & Louw, 1980; Southgate, Masters & Seely, 1996), and could even be carried by the wind from distant locations, where the climatic regimes allow greater primary productivity (Robinson & Seely, 1980). During prolonged periods of drought, with low primary productivity, the dune slip-faces maintain high concentrations of biomass as detritus is deposited by the wind, providing food for detritivores and letting them survive until periods of increased availability of moisture and biomass, when they can proliferate (Southgate, Masters & Seely, 1996).

**Figure 13:**
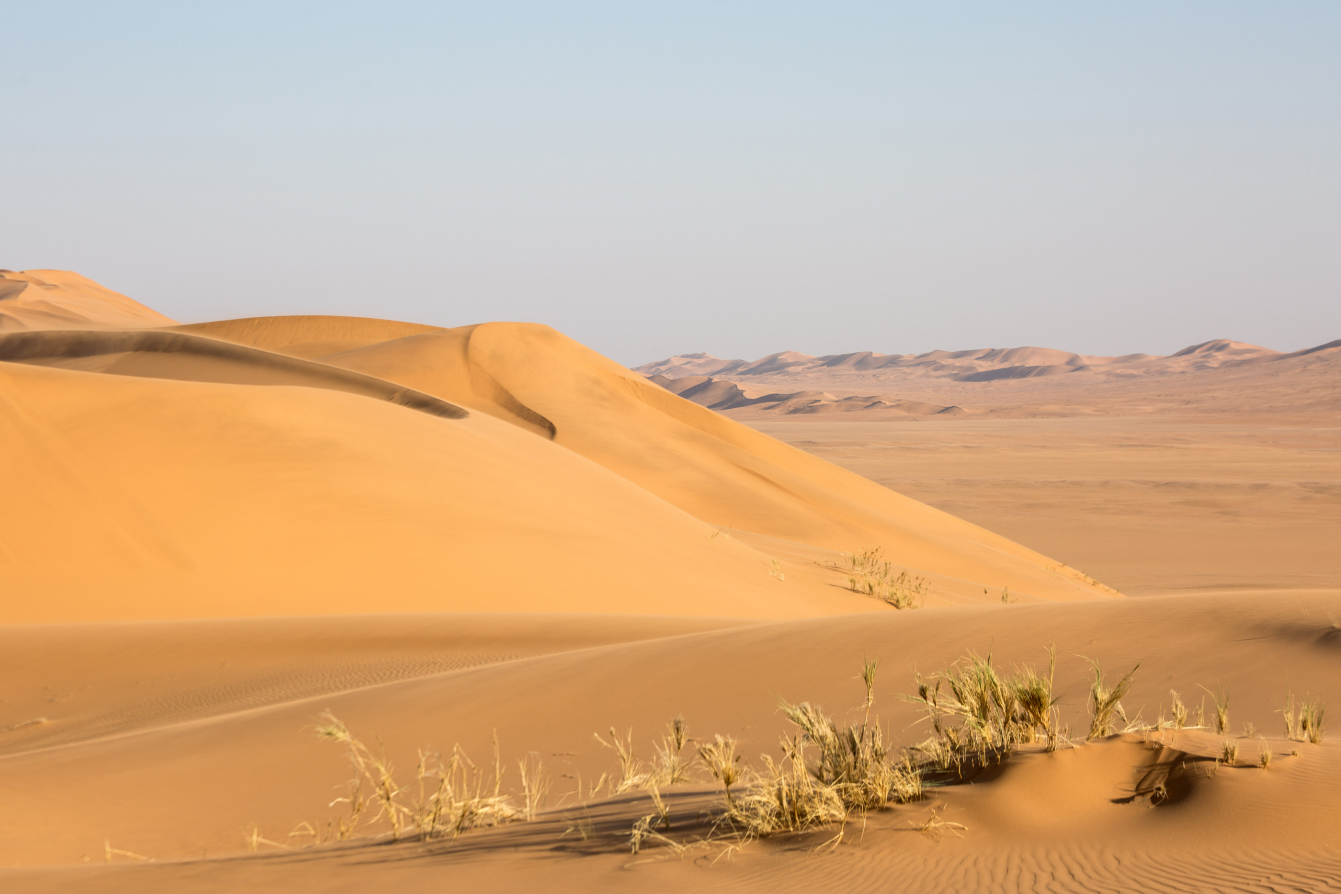
Kahani Dunes in the arid Namib Desert showing some plant growth (*Stipagrostis sabulicola*). Photography courtesy of Oliver Halsey, 2016.

Ornithopods are known to be primary consumers in several Cretaceous ecosystems (Barrett, 2014), therefore, they likely assumed this role in the Botucatu paleodesert as well (Fig. 12F). Based on trackways, Francischini et al. (2015) interpreted the dinosaur fauna of the Botucatu Formation as “dwarf” when compared with the fauna of the older and paleoenvironmentally wetter Guará Formation. It is possible that this could be due to the lower primary productivity of the arid Botucatu paleodesert that would only support viable populations of smaller herbivorous dinosaurs. We infer that the presence of these small-sized herbivorous dinosaurs, less than 68 cm in height to the pelvic girdle, is evidence that there was localized plant growth at least for some period in the region (Fig. 12H to F). This is supported by the fact that the home range of modern herbivorous animals increases with increasing body size (e.g., ungulates: Ofstad et al., 2016), so these small ornithopods with limited home range should not have fed far from where their tracks were found. Therefore, we can infer that the Botucatu paleodesert landscape could have had some shrubs, similar to modern arid dune deserts (e.g., Namib Desert: Fig. 13). The presence of plants makes it reasonable to infer that some invertebrates in the area may not have relied exclusively on detritus to survive but could also have been herbivorous (Fig. 12: flowing energy from H to E).

Contrasting with most “dwarf” trackway producers, a trackway of a large dinosaur (3.6 m high and 5 m long) interpreted as that an ornithopod has been documented as well (Fernandes, 2005; Fernandes & Carvalho, 2007; Francischini et al., 2015). Since the trackway of only one animal was found and there are no trackways of associated predators, it is likely to infer that there was no settled population of this animal in this location at the time. The large ornithopod dinosaur that produced this trackway probably had an extensive home range, encompassing habitats with different primary productivity to support their large body size, and this trackway of an anomalously large animal for the environment would be the record of an animal crossing a less suitable area, like modern desert-dwelling elephants (Viljoen, 1989) and giraffes (Fennessy, 2009; Flanagan et al., 2016).

The next trophic level includes animals from various phylogenetic groups that feed on detritivores and herbivores, therefore, they are carnivores and omnivores (secondary consumers). In the Botucatu Formation, this would include arachnids such as scorpions and spiders (Fig. 12D), the producer of *Paleohelcura araraquarensis* isp. nov. if it had been a predator or an omnivore feeding on animal biomass, small mammaliaform organisms that produced *Brasilichnium elusivum* and *B. saltatorium* (Fig. 12C), and small theropod dinosaurs (Fig. 12B). Due to the high connectivity of the trophic web, there would be interactions among the representatives of this level. Trophic interactions would have been limited by the ability of an animal to prey upon another, which is linked to the size of the prey. Therefore, the animals grouped here probably preyed on smaller animals from the same trophic level or even eggs and juveniles of larger animals. The existence of predatory arachnids in the Botucatu paleodesert does not preclude the existence of predatory insects because this coexistence is observed in modern dune deserts (e.g., Negev: Filser & Prasse, 2008).

*Brasilichnium elusivum* is the most common vertebrate trackway recorded in the Botucatu Formation (Leonardi, Carvalho de & Fernandes, 2007) and is a typical ichnotaxon in other Mesozoic paleodeserts around the world (Krapovickas et al., 2016; Xing et al., 2018). Lizards and snakes that could have occupied the same trophic level are common inhabitants of all modern hot deserts (Cloudsley-Thompson, 1991; Whitford, 2002, p. 129), but no records of these types of animals have been found in the Botucatu Formation. Snakes appeared during the Middle Jurassic (Caldwell et al., 2015), and may not have been widespread in desert ecosystems during the time of the Botucatu paleodesert (Late Jurassic—Early Cretaceous). There are a few records of lizard-like tracks in paleodeserts from the Permian (Loope, 1984; Haubold et al., 1995; Lockley & Hunt, 1995; Lockley et al., 1998). Tracks assigned to lizards (“lacertoids”) generally show digit impressions with an inturned “comb” of curved digits, and pes tracks that are longer than wide (Hunt & Lucas, 2006), however, these were not found in the Botucatu Formation.

The top trophic level is hard to define due to the trophic generalism of desert animals. This level is represented by animals that, because of their size, would be able to prey on non-detritivorous animals, like the producer of *Brasilichnium elusivum*, arachnids and small herbivorous dinosaurs (ornithopods), as well as the small detritivorous animals. Such organisms would include the larger theropod dinosaurs reported from trackways in the Botucatu Formation (Francischini et al., 2015) (Fig. 12B), and mammaliaform producers of *Brasilichnium anaitti*, which are larger than the producers of *Brasilichnium elusivum* and *B. saltatorium* (Fig. 12A).

### Implications for the definition of the *Octopodichnus-Entradichnus* Ichnofacies of eolian environments

Two invertebrate eolian ichnofacies were defined independently, the *Octopodichnus* Ichnofacies of Hunt & Lucas (2007) and the *Entradichnus* Ichnofacies of Ekdale, Bromley & Loope (2007). Subsequently, they were both integrated as the *Octopodichnus*-*Entradichnus* Ichnofacies (Buatois & Mángano, 2011, p. 78; Krapovickas et al., 2016; Buatois & Echevarría, 2019). In particular, Krapovickas et al. (2016) suggested that this ichnofacies is characterized by (1) low to rarely moderate trace fossil diversity, (2) dominance of simple sub-superficial dwelling traces produced mostly by coleopterans, orthopterans and arachnids, with horizontal (e.g., *Palaeophycus*) and/or vertically oriented dwelling burrows (e.g., *Skolithos*, *Digitichnus*); (3) superficial locomotion traces produced by arthropods, especially arachnids (e.g., *Octopodichnus*, *Paleohelcura*); and (4) subordinate simple (*Planolites*) and meniscate (e.g., *Taenidium*, *Entradichnus*) feeding burrows. The *Chelichnus* Ichnofacies of Hunt & Lucas (2007) is considered the archetypal vertebrate ichnofacies of eolian environments. The combined analysis of sedimentary facies and variations in the occurrence, abundance, and diversity of trace fossils may allow differentiation among hyper-arid, arid and semi-arid deserts (Krapovickas et al., 2016).

Like ichnofacies from other continental environments, arid eolian environments display recurrence in the characteristics of their ichnocoenoses, which represent behavioral convergence as a response to abiotic features of habitat and substrate (Buatois & Mángano, 2011). There is also recurrence of certain tracemakers in arid eolian environments, such as scorpions and spiders, which can produce *Paleohelcura* and *Octopodichnus* (Brady, 1947; Davis, Minter & Braddy, 2007), both common ichnotaxa in arid deserts (Krapovickas et al., 2016).

The *Octopodichnus* Ichnofacies is dominated by arthropod trackways and was based on the study of Permian ichnoassemblages, illustrated by the Coconino Sandstone (Hunt & Lucas, 2007). In contrast, the *Entradichnus* Ichnofacies is characterized as dominated by simple shallow vertical and horizontal burrows, as well as meniscate trace fossils, having been based on the study of Jurassic examples, in particular the Navajo Sandstone (Ekdale, Bromley & Loope, 2007). It has been noted that the diverging characterization of these ichnofacies was the result of the disparate databases (Buatois & Mángano, 2011; Krapovickas et al., 2016). Undoubtedly, the apparent contrasting nature of Paleozoic and post-Paleozoic eolian ichnofaunas has been detrimental to a unifying approach to ichnofacies definition. In this regard, the presence of arthropod trackways in Mesozoic eolian deposits (like *P. araraquarensis* isp. nov.) helps to trace a continuity between Paleozoic and post-Paleozoic desert ichnofaunas, further reinforcing the notion of a single *Octopodichnus*-*Entradichnus* Ichnofacies for eolian deposits.

## Conclusions

*Paleohelcura araraquarensis* isp. nov. is characterized by elliptical tracks and a narrow internal width to the trackway. Despite being included in *Paleohelcura*, an ichnogenus usually attributed to arachnids, *Paleohelcura araraquarensis* isp. nov. was most likely produced by a pterygote insect on the basis of neoichnological observations. The producer of *Paleohelcura araraquarensis* isp. nov. could have occupied one of the ecological roles that different species of Pterygota are capable of performing in modern dune deserts.

It could have been a herbivore, or a carnivore (like carnivorous Carabidae in some modern dune deserts) or been part of the fauna of omnivores, being able to adopt herbivorous, carnivorous, and saprophagous diets when opportune. As an omnivore, like the abundant Tenebrionidae beetles in some modern dune deserts, it could have been capable of relying on organic particles that accumulated among the sand grains of the dunes during dry periods with no plant growth. The presence of *Taenidium* isp. and *Skolithos linearis* burrows suggests that the Botucatu paleodesert would have had a detritivorous fauna like modern dune deserts, with the producers of these burrows having a compatible size with the plausible producers of *Paleohelcura araraquarensis* isp. nov.

Based on the interpretation of the ichnofossil producers, it was possible to reconstruct the food web of this paleodesert, in which the producer of *Paleohelcura araraquarensis* isp. nov. could have been a primary consumer if it were a herbivorous or an omnivorous detritivorous insect, or a secondary consumer if it had been produced by predatory insects or omnivores relying on animal biomass. To date, *Paleohelcura araraquarensis* isp. nov. is only known from the Botucatu Formation, but the presence of such types of arthropod trackways in Mesozoic eolian deposits helps to trace a continuity between Paleozoic and post-Paleozoic desert ichnofaunas, further reinforcing the notion of a single *Octopodichnus*-*Entradichnus* Ichnofacies for eolian deposits.

##  Supplemental Information

10.7717/peerj.8880/supp-1 Figure S1 Photographs in original resolution of Work front 1 and 2 in March 2004 depicted in Figure 3

10.7717/peerj.8880/supp-2 Figure S2 Photographs in original resolution of Work front 3 and 4 in March 2004 depicted in Figure 3

10.7717/peerj.8880/supp-3 Figure S3 Track series measured in specimens (A) LPP-IC-0028 (Holotype); (B) LPP-IC-0029 and (C) LPP-IC-0030. The producer walked from bottom to top; Scale bar: 10cm

10.7717/peerj.8880/supp-4 Figure S4 Tracks series measured in specimens (A) LPP-IC-0031; (B) LPP-IC-0032; (C) LPP-IC-0033; (D) LPP-IC-0034; (E) LPP-IC-0035. The producer walked from bottom to top; Scale bar: 10cm

10.7717/peerj.8880/supp-5 Table S1 All measurements taken from the specimens

10.7717/peerj.8880/supp-6 Video S1 Video showing a cockroach (*Blaberus discoidalis*) in ventral view walking on an oiled glass plate Courtesy of R.E. Ritzmann.

## Additional Information and Declarations

### Data Availability

The following information was supplied regarding data availability: The full resolution photographs of the quarry and the work fronts are available in Figs. S1 and S2. Indications of the series measured are available in Figs. S3 and S4. All the measurements taken are available in Table S1. The cockroach video used to produce Fig. 9 is available in Video S1. All the slabs with the ichnofossils (LPP-IC-0028, LPP-IC-0029, LPP-IC-0030, LPP-IC-0031, LPP-IC-0032, LPP-IC-0033, LPP-IC-0034, LPP-IC-0035) are housed in Laboratório de Paleoicnologia e Paleoecologia, Departamento de Ecologia e Biologia Evolutiva, Universidade Federal de São Carlos (UFSCar), São Carlos, São Paulo, Brazil.

### New Species Registration

The following information was supplied regarding the registration of a newly described species: Publication LSID: urn:lsid:zoobank.org:pub:53C73174-4645-40E1-BB7B-75856AEAEAF5 *Paleohelcura araraquarensis* isp. nov. LSID: urn:lsid:zoobank.org:act:7D4303AE-BB63-4474-B79C-AD39AB144917
