# Supplementary figures and images for: A new insect trackway from the Upper Jurassic—Lower Cretaceous eolian sandstones of São Paulo State, Brazil: implications for reconstructing desert paleoecology
Source: PeerJ. 2020 May 22;8:e8880. doi: 10.7717/peerj.8880 (PMC7252435; doi:10.7717/peerj.8880)

Work fronts: 1

2

3

4

South  
←

North  
→

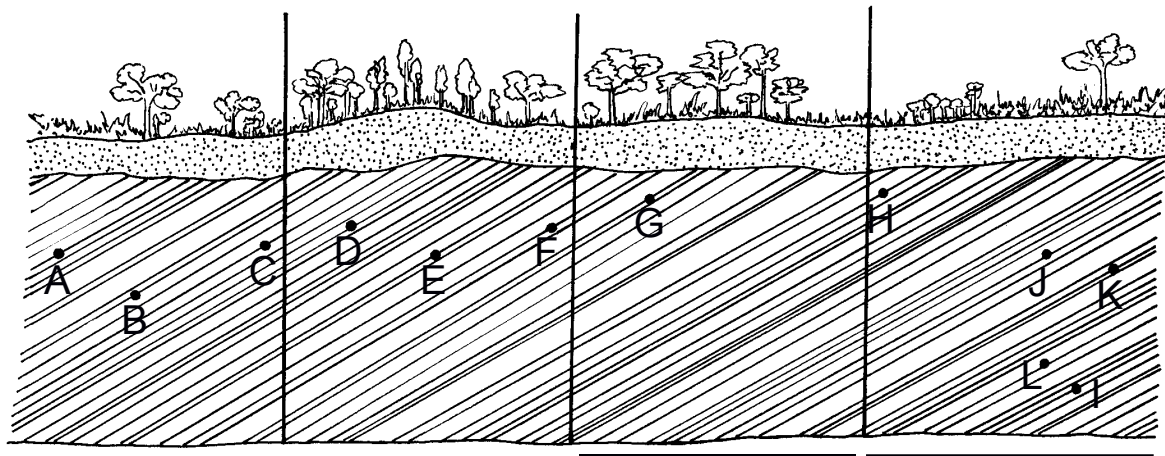

1

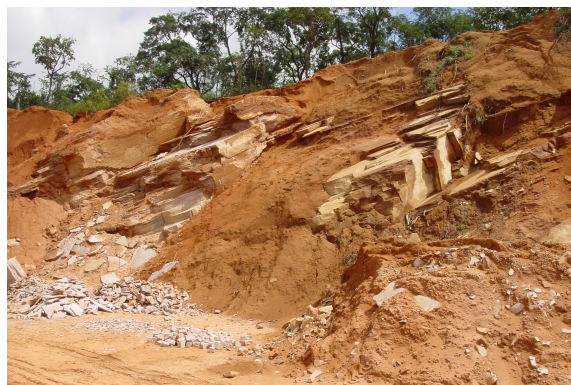

2

3

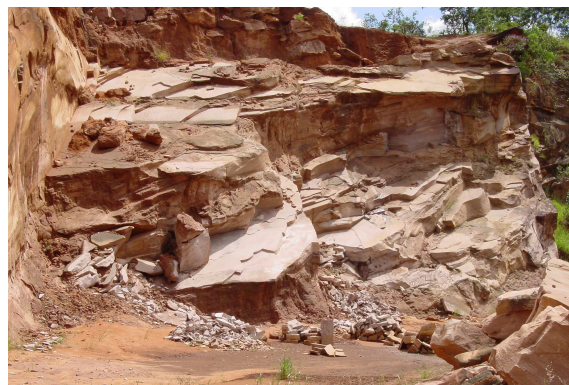

4

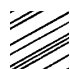

Eolian sandstone

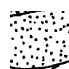

Soil

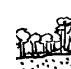

Vegetation

Supplement: Figure S1 [file peerj-08-8880-s001.pdf]

Work fronts: 1

2

3

4

South

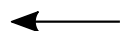

North

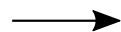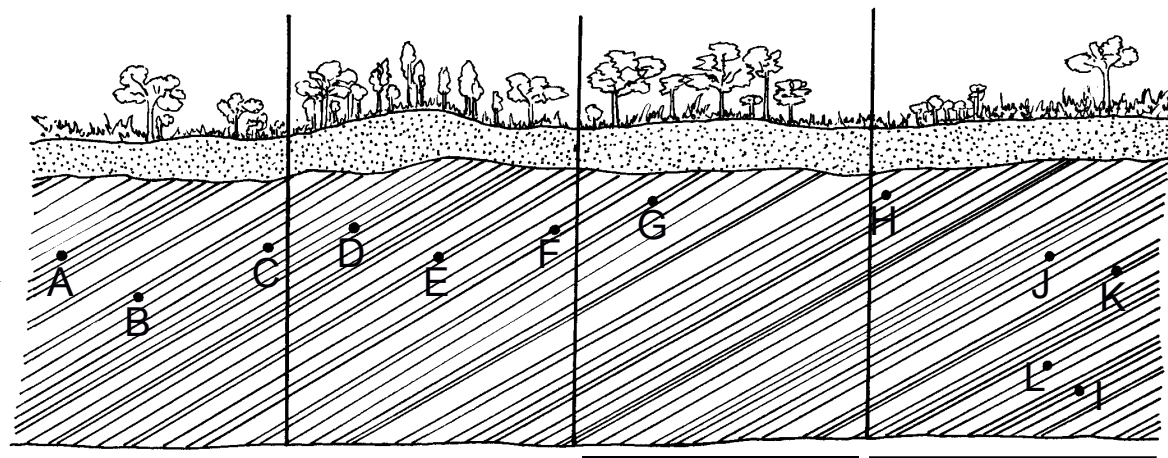

1

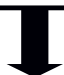

3

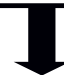

2

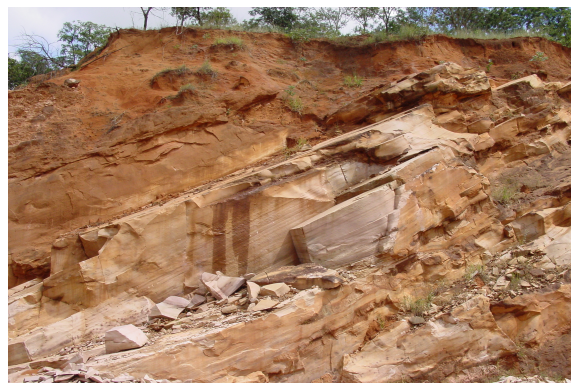

4

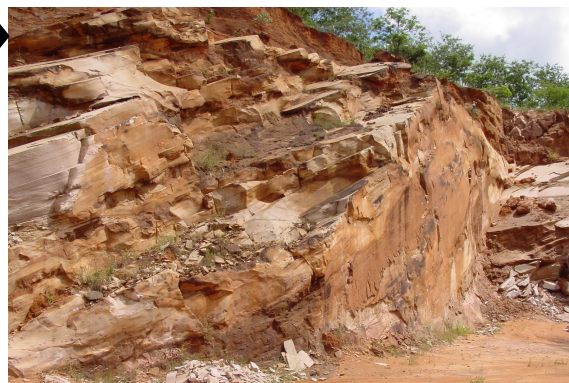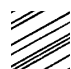

Eolian sandstone

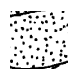

Soil

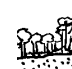

Vegetation

Supplement: Figure S2 [file peerj-08-8880-s002.pdf]

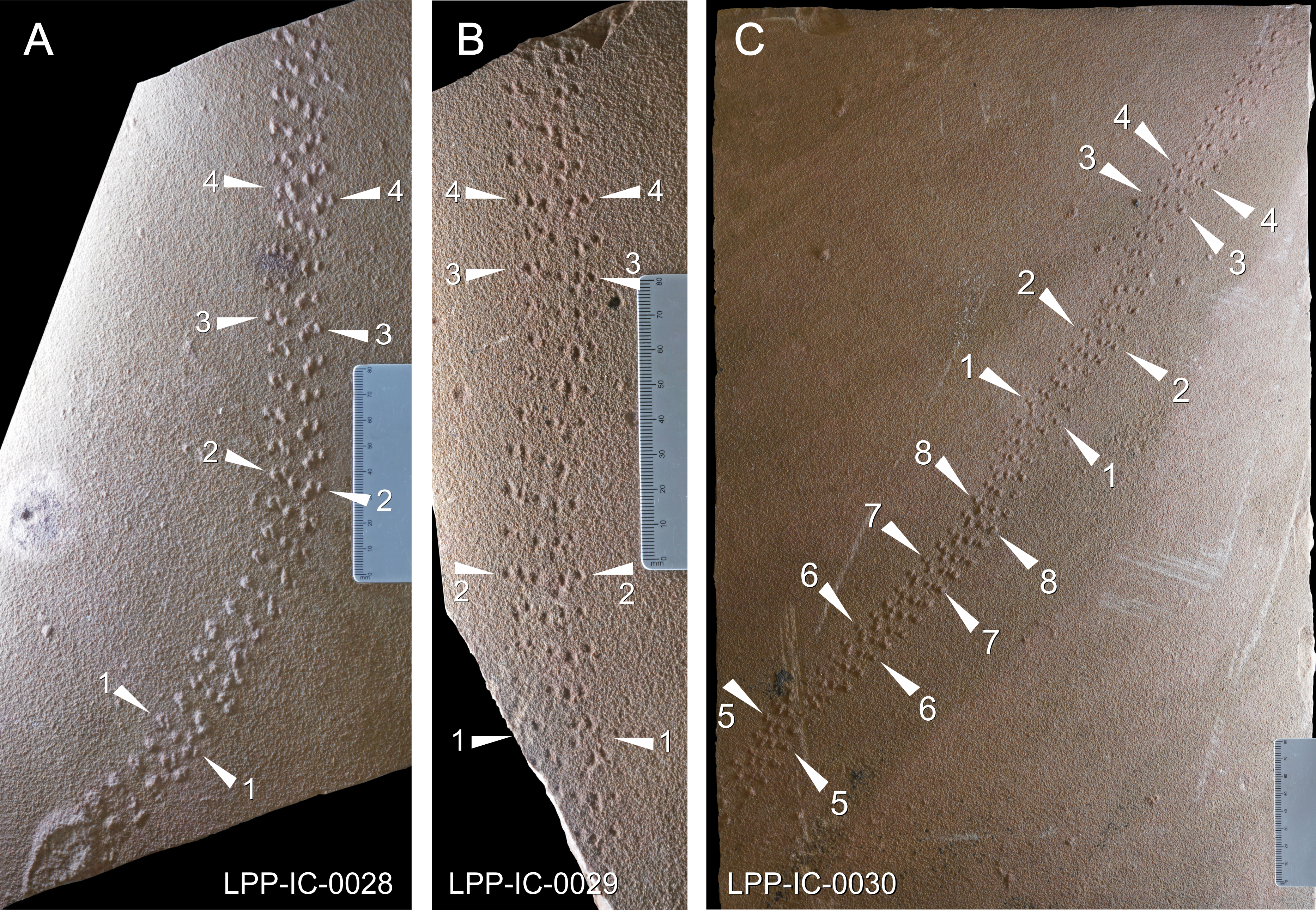

Supplement: Figure S3 [file peerj-08-8880-s003.jpg]

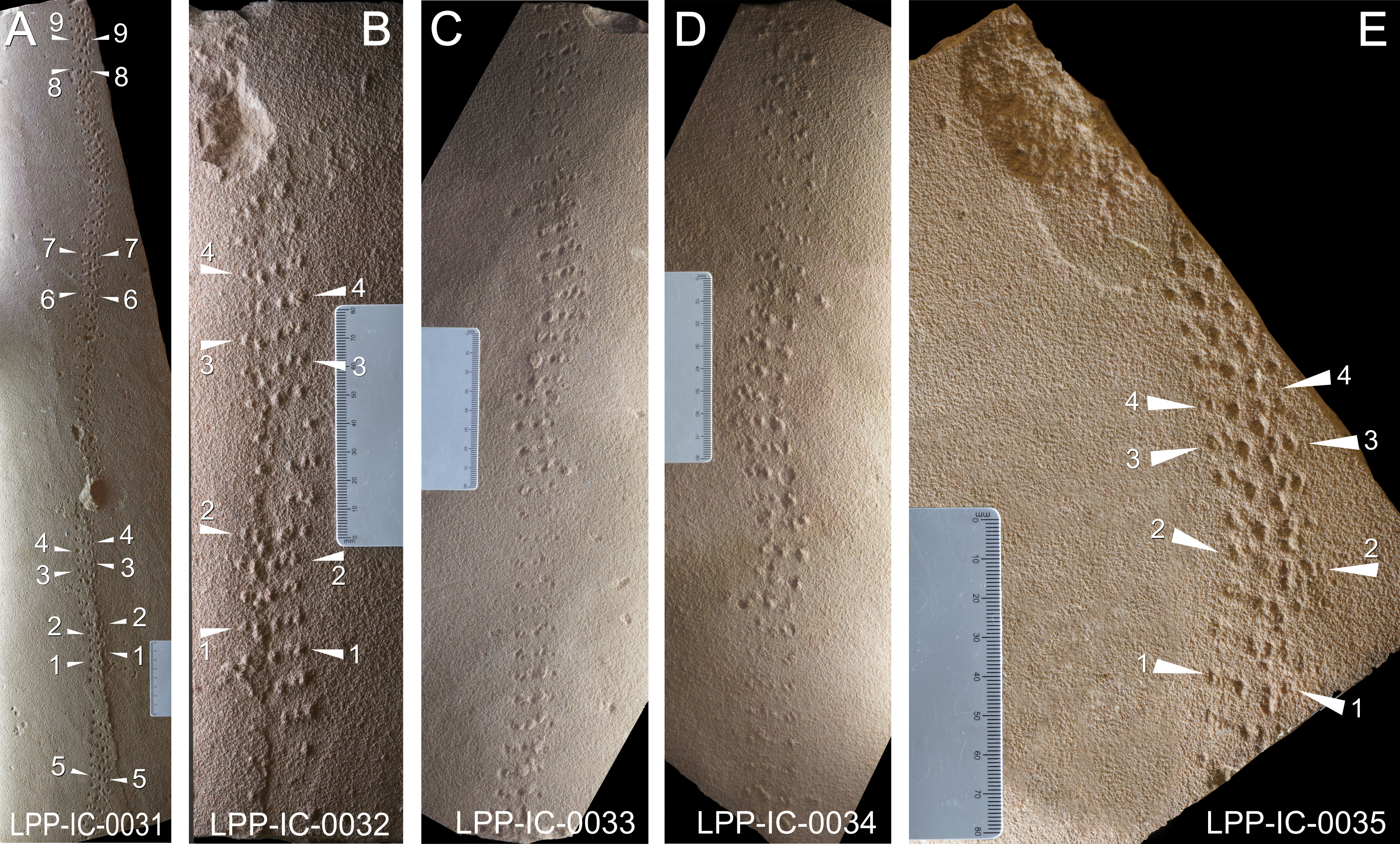

Supplement: Figure S4 [file peerj-08-8880-s004.jpg]
